# Metabolic and Stress Response Changes Precede Disease Onset in the Spinal Cord of Mutant SOD1 ALS Mice

**DOI:** 10.3389/fnins.2019.00487

**Published:** 2019-05-31

**Authors:** Gavin Pharaoh, Kavithalakshmi Sataranatarajan, Kaitlyn Street, Shauna Hill, Jake Gregston, Bumsoo Ahn, Caroline Kinter, Michael Kinter, Holly Van Remmen

**Affiliations:** ^1^Aging and Metabolism Research Program, Oklahoma Medical Research Foundation, Oklahoma City, OK, United States; ^2^Department of Physiology, University of Oklahoma Health Science Center, Oklahoma City, OK, United States; ^3^Department of Cellular and Structural Biology, University of Texas Health Science Center at San Antonio, San Antonio, TX, United States; ^4^Oklahoma City VA Medical Center, Oklahoma City, OK, United States

**Keywords:** amyotrophic lateral sclerosis (ALS), SOD1^G93A^ ALS mouse model, metabolism, hypermetabolism, mitochondria, antioxidants, stress response, mitochondrial unfolded protein response

## Abstract

Many Amyotrophic Lateral Sclerosis (ALS) patients experience hypermetabolism, or an increase in measured vs. calculated metabolic rate. The cause of hypermetabolism and the effects on neuronal metabolism in ALS are currently unknown, but the efficacy of dietary interventions shows promise for metabolism as an ALS therapeutic target. The goal of this study is to measure changes in metabolic pathways as a function of disease progression in spinal cords of the SOD1^G93A^ mouse model of ALS. We conducted a comprehensive assessment of protein expression for metabolic pathways, antioxidants, chaperones, and proteases in lumbar spinal cord from male SOD1^G93A^ mice at pre-onset, onset, and end-stages of the disease using targeted proteomic analysis. These results reveal that protein content of metabolic proteins including proteins involved in glycolysis, β-oxidation, and mitochondrial metabolism is altered in SOD1^G93A^ mouse spinal cord well before disease onset. The changes in mitochondrial metabolism proteins are associated with decreased maximal respiration and glycolytic flux in SOD1^G93A^ dermal fibroblasts and increased hydrogen peroxide and lipid hydroperoxide production in mitochondria from sciatic nerve and gastrocnemius muscle fibers at end stage of disease. Consistent with redox dysregulation, expression of the glutathione antioxidant system is decreased, and peroxiredoxins and catalase expression are increased. In addition, stress response proteases and chaperones, including those involved in the mitochondrial unfolded protein response (UPR^mt^), are induced before disease onset. In summary, we report that metabolic and stress response changes occur in SOD1^G93A^ lumbar spinal cord before motor symptom onset, and are primarily caused by SOD1^G93A^ expression and do not vary greatly as a function of disease course.

## Introduction

Amyotrophic Lateral Sclerosis (ALS) is a cell non-autonomous disease affecting motor neurons as well as surrounding support cells including astrocytes, oligodendrocytes, and microglia (Ilieva et al., 2009; Rizzo et al., 2014) and skeletal muscle (Dobrowolny et al., 2008). Motor neuron cell death causes loss of neuromuscular innervation resulting in severe muscle atrophy and paralysis culminating in death by respiratory failure (Rowland and Shneider, 2001). While recent advances in sequencing technologies have led to the identification of genes underlying over 60% of familial cases (fALS) and over 10% of sporadic cases (sALS), the pathogenesis of ALS is still poorly understood delaying the development of effective treatments (Renton et al., 2014). More importantly, the only FDA approved drugs for the treatment of ALS, riluzole and edaravone, provide only minor improvements for ALS patients (Zoccolella et al., 2007; Abe et al., 2014).

The first mutated gene identified in fALS was superoxide dismutase 1 (*Sod1*), an antioxidant enzyme that detoxifies superoxide. Several transgenic mouse lines expressing mutant *Sod1*, including SOD1^G93A^, have been generated that recapitulate fALS symptoms and have allowed exploration of biochemical mechanisms underlying fALS pathogenesis (Ilieva et al., 2009). One phenotype of SOD1 mutant mice that also recapitulates the human disease is a hypermetabolic phenotype that occurs before the onset of motor symptoms (Dupuis et al., 2004). Approximately 50% of ALS patients experience the phenomenon of hypermetabolism, wherein their measured resting energy expenditure is increased compared to their calculated resting energy expenditure (Bouteloup et al., 2009). Patients rarely switch metabolic groups during disease progression, and the hypermetabolism phenotype does not correlate with fat free mass, age, sex, BMI, or an ALS functional test (Bouteloup et al., 2009). However, hypermetabolic patients display a trend toward decreased survival length from symptom onset (Bouteloup et al., 2009), and a phase II clinical trial suggested that high calorie diets had beneficial survival effects in ALS patients (Wills et al., 2014). In support of this, a high calorie diet extends SOD1 mutant mouse lifespan (Dupuis et al., 2004), while caloric restriction reduced lifespan (Hamadeh et al., 2005). The hypermetabolism phenotype is a potential disease modifier that can be targeted to affect patient disease progression.

We hypothesized that increased energy demands (hypermetabolism) cause an energetic stress affecting metabolism in motor neurons that contributes to disease progression. To test our hypothesis, we collected lumbar spinal cord from SOD1^G93A^ mice and age-matched wildtype controls before and at onset of motor symptoms and at disease end-stage. We measured changes in protein abundance of major metabolic pathway enzymes at all three disease stages via a targeted proteomics approach. In addition, because mitochondrial damage occurs early in ALS pathogenesis, we measured sciatic nerve and skeletal muscle mitochondrial function and lipid hydroperoxide and hydrogen peroxide production. We also measured expression of spinal cord and skeletal muscle mitochondrial proteases and chaperones, including those involved in the mitochondrial unfolded protein response (UPR^mt^), a stress response pathway responsible for maintaining mitochondrial protein quality control in response to accumulation of misfolded or unfolded proteins in the mitochondria (Hill and Van Remmen, 2014). Our results reported here represent the first comprehensive measurement of major metabolic and stress response pathways in spinal cord of ALS mutant mice as a function of disease stage.

## Results

### Metabolic Pathways Are Differentially Regulated in the Spinal Cord of SOD1^G93A^ Mice, and Stress Response Proteins Are Induced

**FIGURE 1:**
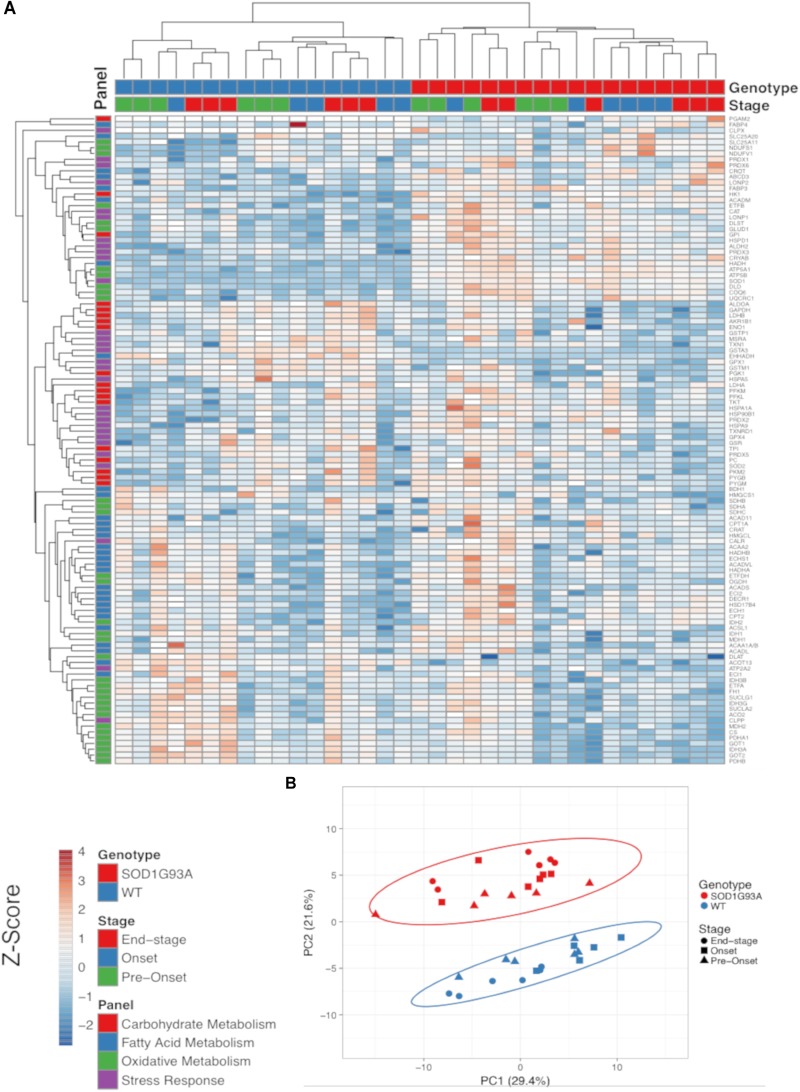
Metabolic pathways are differentially regulated in the spinal cord of SOD1^G93A^ mice, and stress response proteins are induced. Protein content from spinal cords of pre-onset, onset, and end-stage control and SOD1^G93A^ mice (*n* = 5–6) measured by mass spectrometry. The assayed proteins were targeted for representative panels of carbohydrate metabolism, fatty acid metabolism, oxidative mitochondrial metabolism (tricarboxylic acid cycle and electron transport chain), and stress response (antioxidants, chaperones, heat shock proteins, and proteases). **(A)** Heatmap with clustering for each sample using a Z-score scale. **(B)** Principal component analysis (PCA) plot for principal components 1 and 2. Protein names, IDs, and absolute expression values are detailed in Supplemental Table 1.

The SOD1^G93A^ ALS mouse model recapitulates the hypermetabolism phenotype (Dupuis et al., 2004). In addition to the well characterized decline in body and muscle mass, we also observed reduced fat mass in end-stage SOD1^G93A^ mice (Supplemental Figure 1). Because ALS is a neurodegenerative disease characterized by pathological changes in the spinal cord, we used targeted proteomic analysis to measure differential protein content of metabolic and stress response pathways in spinal cord homogenates from wild-type and SOD1^G93A^ mice over the time course of the disease. We interrogated changes in 113 proteins detected in at least one genotype (Figure 1 and Supplemental Table 1). We categorized proteins into panels for carbohydrate metabolism, fatty acid metabolism, oxidative mitochondrial metabolism [the tricarboxylic acid cycle and electron transport chain (ETC)], and stress response (antioxidants, chaperones, heat shock proteins, and proteases). Metabolic and stress response protein expression differences in SOD1^G93A^ lumbar spinal cord are evident throughout disease course (Figure 1A). Principal component analysis (PCA) demonstrates that protein expression in lumbar spinal cord from SOD1^G93A^ mice clusters distinctly from spinal cord from control mice beginning before onset of symptoms, but no separation occurs in clustering between disease stages (Figure 1B). 49% of assayed proteins exhibit a significant difference due to genotype effect, while only 10% exhibited a significant difference based on disease stage using two-way ANOVA corrected for false discovery rate with the Benjamini-Hochberg procedure. These results show that metabolism and stress response protein content is already dramatically altered in spinal cords from SOD1^G93A^ mice before onset of motor symptoms.

#### Glycolysis and the Malate-Aspartate Shuttle

**FIGURE 2:**
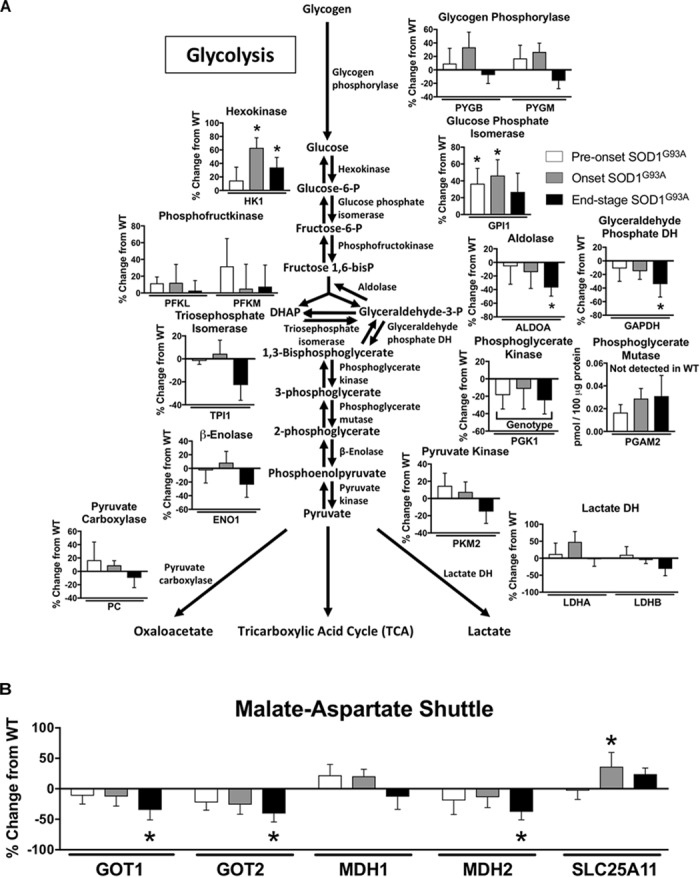
Glycolysis and the malate-aspartate shuttle. Changes in protein content of carbohydrate metabolism in SOD1^G93A^ spinal cords compared to controls measured by mass spectrometry (*n* = 5–6). **(A)** Glycolysis. **(B)** Malate-aspartate shuttle. Data are represented as percent change relative to the same time point wild-type control. Bars represent means ± standard deviation. Phosphoglycerate mutase 2 (PGAM2) did not reach the level of detection in wild-type samples but did in SOD1^G93A^. “Genotype” denotes a significant genotype effect in Benjamini-Hochberg corrected two-way ANOVA with *p* < 0.05 but no individual Multiple Comparison test was significant. Significance symbols demonstrate *p* < 0.05 for a genotype effect and additional significant results for the Tukey Multiple Comparison test. ^∗^*p* < 0.05 wild-type vs. SOD1^G93A^ at the designated time point. SOD1^G93A^ pre-onset (white), SOD1^G93A^ onset (gray), SOD1^G93A^ end-stage (black). DH, dehydrogenase; DHAP, Dihydroxyacetone phosphate. Protein names, IDs, and absolute expression values are detailed in Supplemental Table 1.

Two key proteins involved in glycolysis are elevated prior to disease onset, but we also observe a decline at end-stage in several glycolytic proteins along with a decline in some key proteins involved in the malate aspartate shuttle in spinal cord from SOD1^G93A^ mice Specifically, levels for the first two enzymes in glycolysis (HK1 and GPI1) are increased beginning at pre-onset or disease onset. However, ALDOA and GAPDH are reduced at end-stage and PGK1 exhibits a genotype specific reduction in spinal cords from SOD1^G93A^ mice compared to wild-type controls (Figure 2A). PGAM2 only reached the threshold of detection in SOD1^G93A^ samples (Figure 2A). At end-stage, protein content of key components of the malate-aspartate shuttle declines (Figure 2B). While SLC25A11, the mitochondrial inner-membrane α-ketoglutarate/malate carrier, is increased at onset, at end-stage the cytoplasmic and mitochondrial aspartate aminotransferases (GOT1 and GOT2) and the mitochondrial malate dehydrogenase (MDH2) are decreased compared to wild-type controls (Maglott et al., 2011; Figure 2B). TKT, a key enzyme in the pentose-phosphate pathway is increased in spinal cords from SOD1^G93A^ mice (Supplemental Table 1). Combined these results suggest altered carbohydrate utilization in end-stage spinal cords from SOD1^G93A^ mice compared to wild-type control mice.

#### β-Oxidation

**FIGURE 3:**
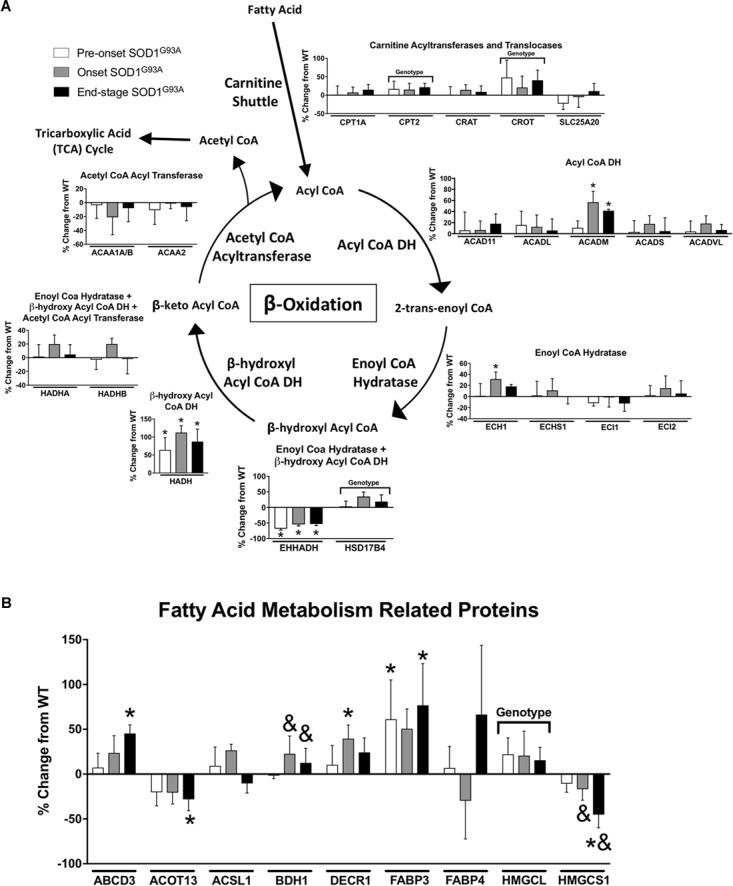
Fatty acid metabolism. Changes in protein content of fatty acid metabolism in SOD1^G93A^ spinal cords compared to controls measured by mass spectrometry (*n* = 5–6). **(A)** β-oxidation. **(B)** Fatty acid metabolism related proteins. Data are represented as percent change relative to the same time point wild-type control. Bars represent means ± standard deviation. “Genotype” denotes a significant genotype effect in Benjamini-Hochberg corrected two-way ANOVA with *p* < 0.05 but no individual Multiple Comparison test was significant. Significance symbols demonstrate *p* < 0.05 for a genotype effect and additional significant results for the Tukey Multiple Comparison test. ^∗^*p* < 0.05 wild-type vs. SOD1^G93A^ at the designated time point; ^&^*p* < 0.05 vs. SOD1^G93A^ pre-onset. SOD1^G93A^ pre-onset (white), SOD1^G93A^ onset (gray), SOD1^G93A^ end-stage (black). DH, dehydrogenase; CoA, coenzyme A. Protein names, IDs, and absolute expression values are detailed in Supplemental Table 1.

Several proteins involved in mitochondrial β-oxidation are increased in spinal cords from SOD1^G93A^ mice. CPT2, which is involved in import of fatty acids to mitochondria, and CROT, which is involved in export of β-oxidation products from peroxisomes, are both increased in SOD1^G93A^ samples (Figure 3A). The mitochondrial medium chain acyl-CoA dehydrogenase (ACADM) is increased at onset and end-stage in spinal cord from SOD1^G93A^ mice compared to wild-type mice, and the mitochondrial β-hydroxyl acyl CoA dehydrogenase (HADH) is increased throughout the disease course in SOD1^G93A^ mice (Figure 3A). Enoyl-CoA Hydratase 1 (ECH1), which is targeted to both mitochondria and peroxisomes, and DECR1, an auxiliary enzyme of mitochondrial β-oxidation are increased at disease onset in SOD1^G93A^ mice (Figure 3A,B). The fatty acid transporters ABCD3 and FABP3 are upregulated beginning at disease onset (Figure 3B). In the peroxisome, the peroxisomal bifunctional enzyme HSD17B4 is increased in spinal cord from SOD1^G93A^ while EHHADH is decreased throughout disease course (Figure 3A).

Several proteins involved in other aspects of fatty acid metabolism are differentially regulated. BDH1 is a mitochondrial dehydrogenase that interconverts two ketone bodies produced during fatty acid catabolism (Maglott et al., 2011) and is increased in onset and end-stage compared to pre-onset in spinal cords from SOD1^G93A^ mice (Figure 3B). ACOT13 is a mitochondrial medium- and long-chain acyl-CoA thioesterase and is decreased at end-stage compared to wild-type (Figure 3B). HMGCS1 catalyzes production of HMG-CoA, an intermediate in ketogenesis and isoprenoid biosynthesis, including synthesis of a wide variety of molecules such as cholesterol, ubiquinone, and steroid hormones (Holstein and Hohl, 2004; Maglott et al., 2011). HMGCS1 expression is decreased beginning at disease onset in SOD1^G93A^ mice (Figure 3B). HMGCL, a key enzyme in ketogenesis, is increased in SOD1^G93A^ spinal cords. Taken together, these data suggest an increase in mitochondrial fatty acid metabolism in ALS spinal cords.

#### Tricarboxylic Acid (TCA) Cycle

**FIGURE 4:**
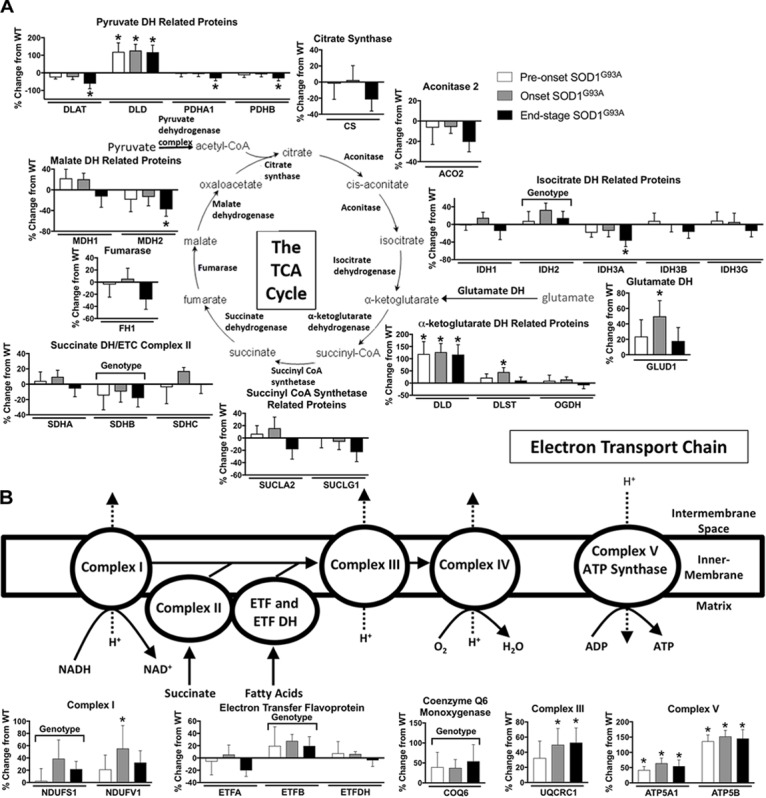
Oxidative mitochondrial metabolism. Changes in protein content of oxidative mitochondrial metabolism in SOD1^G93A^ spinal cords compared to controls measured by mass spectrometry (*n* = 5–6). **(A)** The tricarboxylic acid (TCA) cycle. **(B)** The electron transport chain (ETC). Data are represented as percent change relative to the same time point wild-type control. Bars represent means ± standard deviation. “Genotype” denotes a significant genotype effect in Benjamini-Hochberg corrected two-way ANOVA with *p* < 0.05 but no individual Multiple Comparison test was significant. Significance symbols demonstrate *p* < 0.05 for a genotype effect and additional significant results for the Tukey Multiple Comparison test. ^∗^*p* < 0.05 wild-type vs. SOD1^G93A^ at the designated time point. SOD1^G93A^ pre-onset (white), SOD1^G93A^ onset (gray), SOD1^G93A^ end-stage (black). DH, dehydrogenase; CoA, coenzyme A; ETC, electron transport chain; ETF, electron transfer flavoprotein. Protein names, IDs, and absolute expression values are detailed in Supplemental Table 1.

TCA proteins are generally decreased at end-stage in spinal cords from SOD1^G93A^ mice. Three subunits of pyruvate dehydrogenase (DLAT, PDHA1, PDHB), IDH3A, and MDH2 are decreased in end-stage in spinal cords from SOD1^G93A^ mice compared to wild-type control mice (Figure 4A). However DLD, the E3 component of α-ketoglutarate dehydrogenase is increased throughout disease course. Another subunit of α-ketoglutarate dehydrogenase, DLST, and glutamate dehydrogenase (GLUD1) are increased at onset suggesting increased entry of substrates into the TCA cycle from the glutamate pathway and decreased entry from carbohydrate metabolism (Figure 4A).

#### Mitochondrial Electron Transport Chain (ETC)

The content of several assayed proteins in the mitochondrial ETC is increased in spinal cord from the SOD1^G93A^ mice. Subunits of ETC Complex I (NDUFS1, NDUFV1), Complex III (UQCRC1), and Complex V (ATP5A1, ATP5B), as well as ETF (ETFB) are increased in SOD1^G93A^ spinal cord beginning before disease onset (Figure 4B). COQ6, which performs a biosynthetic step of ubiquinone synthesis, is also increased in SOD1^G93A^ spinal cord. In contrast, one subunit of Complex II (SDHB) is decreased (Figure 4B). Because we saw increased expression of ETC proteins in spinal cords from SOD1^G93A^ mice, we assessed gene expression for nuclear- and mitochondrial-encoded ETC subunits and PGC1α, a transcription factor involved in mitochondrial biogenesis (Scarpulla, 2011), in spinal cords and gastrocnemius muscles from SOD1^G93A^ mice. Expression of all assayed ETC complex subunits is increased and all exhibit a significant difference by genotype effect in spinal cords from SOD1^G93A^ mice compared to controls (Supplemental Table 3 and Supplemental Figure 2A). However, no difference due to genotype is observed in PGC1α expression. In gastrocnemius muscle, no difference is identified between genotypes for ETC subunit expression, but *Ppargc1a* (PGC1α) expression is reduced at end-stage compared to wild-type controls (Supplemental Table 4 and Supplemental Figure 2B).

#### Antioxidant Enzymes

**FIGURE 5:**
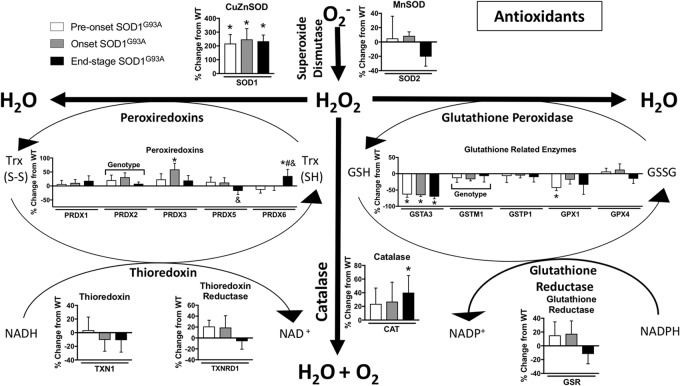
Antioxidants. Changes in protein content of major antioxidants in SOD1^G93A^ spinal cords compared to controls measured by mass spectrometry (*n* = 5–6). Data are represented as percent change relative to the same time point wild-type control. Bars represent means ± standard deviation. “Genotype” denotes a significant genotype effect in Benjamini-Hochberg corrected two-way ANOVA with *p* < 0.05 but no individual Multiple Comparison test was significant. Significance symbols demonstrate *p* < 0.05 for a genotype effect and additional significant results for the Tukey Multiple Comparison test. ^∗^*p* < 0.05 wild-type vs. SOD1^G93A^ at the designated time point; ^&^*p* < 0.05 vs. SOD1^G93A^ pre-onset; ^#^*p* < 0.05 vs. SOD1^G93A^ onset. SOD1^G93A^ pre-onset (white), SOD1^G93A^ onset (gray), SOD1^G93A^ end-stage (black). O_2_^-^, superoxide; H_2_O_2_, hydrogen peroxide; GSH, reduced glutathione; GSSG, oxidized glutathione disulfide; TRX (SH), reduced thioredoxin; TRX (S-S), oxidized thioredoxin. Protein names, IDs, and absolute expression values are detailed in Supplemental Table 1.

Oxidative stress has been implicated as an important underlying factor in ALS pathogenesis (Barber and Shaw, 2010). We found many changes in the expression of hydrogen peroxide detoxification enzymes in spinal cord from SOD1^G93A^ mice compared to wild-type mice. The glutathione system, peroxiredoxins, and catalase are altered in spinal cords from SOD1^G93A^ mice (Figure 5). PRDX2, PRDX3, CAT, and PRDX6 are increased in SOD1^G93A^ compared to wild-type control (Figure 5). Simultaneously, GPX1 (glutathione peroxidase 1), GSTM1, and GSTA3 are decreased in spinal cords from SOD1^G93A^ mice (Figure 5). SOD1 is upregulated in SOD1^G93A^ compared to wild-type control, validating SOD1 overexpression in the spinal cord samples, while no change is observed in SOD2, the mitochondrial superoxide dismutase (Figure 5).

#### Stress Response Proteases and Chaperones

**FIGURE 6:**
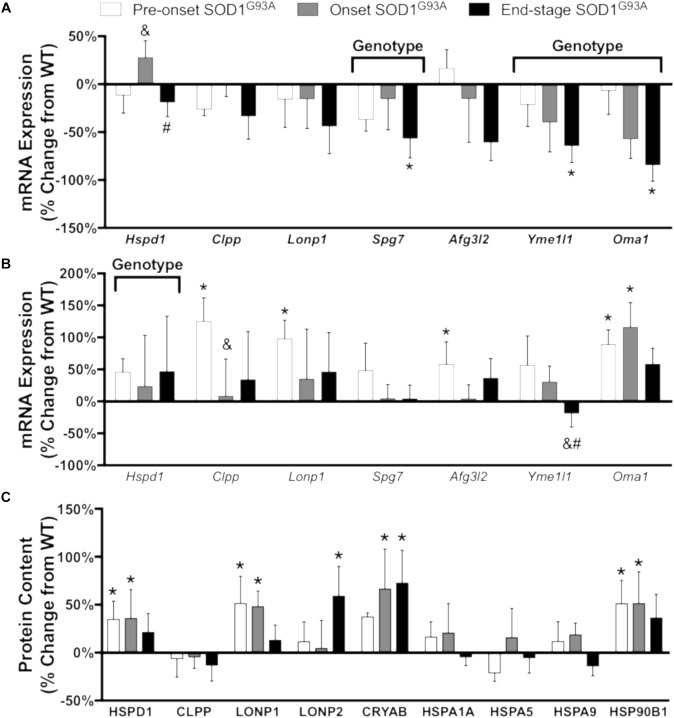
Chaperones and proteases. Changes in mRNA expression and protein content of chaperones and proteases in SOD1^G93A^ gastrocnemius muscle and spinal cords compared to wild-type controls measured by RT-PCR and mass spectrometry (*n* = 5–6). **(A)** mRNA expression in the gastrocnemius muscle. **(B)** mRNA expression in the spinal cord. **(C)** Protein content in the spinal cord. Data are represented as percent change relative to the same time point wild-type control. Bars represent means ± standard deviation. “Genotype” denotes a significant genotype effect in Benjamini-Hochberg corrected two-way ANOVA with *p* < 0.05 but no individual Multiple Comparison test was significant. Significance symbols demonstrate *p* < 0.05 for a genotype effect and additional significant results for the Tukey Multiple Comparison test. ^∗^*p* < 0.05 wild-type vs. SOD1^G93A^ at the designated time point; ^&^*p* < 0.05 vs. SOD1^G93A^ pre-onset; ^#^*p* < 0.05 vs. SOD1^G93A^ onset. SOD1^G93A^ pre-onset (white), SOD1^G93A^ onset (gray), SOD1^G93A^ end-stage (black). Gene IDs and relative expression (RQ) values are detailed in Supplemental Tables 3 (spinal cord) and 4 (gastrocnemius). Protein names, IDs, and absolute expression values are detailed in Supplemental Table 1.

Misfolding and accumulation of ubiquitinated cytoplasmic mutant SOD1 inclusions has been observed early in disease progression and likely plays a role in pathogenesis (Ilieva et al., 2009). To understand the cellular response to accumulation of these aggregates, we measured the mRNA expression and protein content of a panel of proteases and chaperones. In spinal cord, mRNA expression of the mitochondrial proteases *Clpp, Lonp1, Afg3l2*, and *Oma1* is increased pre-onset, *Yme1l1* is decreased at end-stage, and the chaperone *Hspd1* is increased (Figure 6B and Supplemental Table 3). While CLPP is not increased at the protein level, CLPX, the chaperone subunit of the CLPX/CLPP oligomer, only reached the limit of detection in spinal cords from SOD1^G93A^ mice (Supplemental Table 1). In addition, protein expression of LONP1 and LONP2 as well as the heat shock proteins HSPD1 (Hsp60), CRYAB, and HSP90B1 is increased in spinal cords from SOD1^G93A^ mice compared to controls beginning before disease onset (Figure 6C). In contrast, mRNA expression of *Hspd1, Spg7, Yme1l1*, and *Oma1* is decreased in gastrocnemius at end-stage (Figure 6A and Supplemental Table 4). ALDH2, the mitochondrial aldehyde dehydrogenase, protects against oxidative stress (Ohta et al., 2004) and is upregulated in onset SOD1^G93A^ compared to wild-type control (Supplemental Table 1). ATP2A2, an endoplasmic reticulum calcium pump, is decreased at end-stage (Supplemental Table 1). Together, these data suggest that regulation of stress response to SOD1^G93A^ expression is tissue specific, and spinal cords from SOD1^G93A^ mice experience an increase beginning before onset of motor symptoms. mRNA expression or protein content of 4 proteins (HSPD1, CLPP, LONP1, and CLPX) involved in the UPR^mt^ was increased in spinal cords but not muscle from SOD1^G93A^ mice.

### Mitochondrial Function in Cultured Fibroblasts, Sciatic Nerve Tissue, and Permeabilized Muscle Fibers of SOD1^G93A^ Mice

**FIGURE 7:**
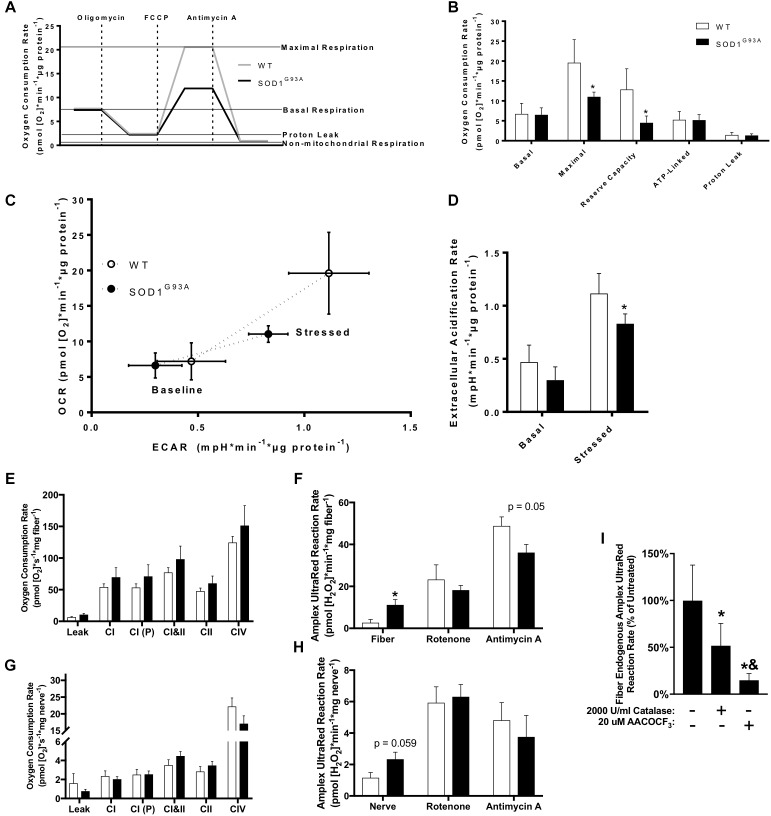
Mitochondrial function in cultured fibroblasts, sciatic nerve tissue, and permeabilized muscle fibers of SOD1^G93A^ mice. **(A)** Schematic showing the output from Seahorse XF24 Flux Analyzer and calculation of measurements. **(B)** Oxygen consumption rate (OCR), **(C)** Extracellular acidification rate (ECAR), and **(D)** Comparison of OCR and ECAR of dermal fibroblasts isolated from wild-type (WT) and SOD1^G93A^ mice (*n* = 5) measured by Seahorse XF24 Flux Analyzer. Stressed ECAR was measured in the presence of oligomycin and FCCP. Simultaneous OCR and hydrogen peroxide and lipid hydroperoxide production rates (Amplex UltraRed) were assessed in permeabilized sciatic nerves (*n* = 8–11) and red gastrocnemius fiber biopsies (*n* = 7–9) from end-stage female mice using the OROBOROS Oxygraph-2k with fluorometer. **(E)** Gastrocnemius fiber OCR. **(F)** Gastrocnemius fiber hydrogen peroxide and lipid hydroperoxide production rates. **(G)** Sciatic nerve OCR. **(H)** Sciatic nerve hydrogen peroxide and lipid hydroperoxide production rates. ^∗^*p* < 0.05 wild-type vs. SOD1^G93A^ determined by unpaired student’s *t*-test. **(I)** Basal hydrogen peroxide and lipid hydroperoxide production rates for SOD1^G93A^ gastrocnemius fibers in the OROBOROS O2K fluorometer vehicle treated, or pre-treated with 2000 U/ml Catalase or 20 μM AACOCF_3_ (*n* = 7). ^∗^*p* < 0.05 treated (either catalase or AACOCF_3_) vs. untreated control, ^&^*p* < 0.05 catalase vs. AACOCF_3_ treated. Substrate-inhibitor protocol and definitions of states are described in Materials and Methods. Bars represent means ± standard deviation. Wild-type (white) and SOD1^G93A^ (black). CI, mitochondrial Complex I (with glutamate, malate, and ADP); CI(P), mitochondrial Complex I (with glutamate, malate, pyruvate, and ADP); CII, mitochondrial Complex II; CIV, mitochondrial Complex IV; FCCP, p-trifluoromethoxy carbonyl cyanide phenyl hydrazone; AACOCF_3_, Arachidonyl trifluoromethyl ketone.

Mitochondrial dysfunction has been broadly implicated in the pathogenesis of neurodegenerative diseases including ALS (Schon and Manfredi, 2003), and impaired mitochondrial function is observed in human fALS SOD1 mutant cultured fibroblasts (Allen et al., 2014). We analyzed oxygen consumption rate (OCR) in pre-onset SOD1^G93A^ mouse dermal fibroblasts with the Seahorse XF24 using sequential additions of the Complex V/ATP synthase inhibitor oligomycin, protonophore FCCP, and Complex III inhibitor antimycin A to measure several parameters of mitochondrial function (Figure 7A). SOD1^G93A^ mouse fibroblasts have reduced maximal respiration and reserve capacity compared to wild-type controls, but no change in basal or ATP-linked respiration and no change in proton leak (Figure 7B). Extracellular acidification rate (ECAR) is a measurement of glycolysis using change in pH of the media (Wu et al., 2007). ECAR is non-significantly decreased at baseline but is significantly reduced when stressed with oligomycin and FCCP in SOD1^G93A^ mouse fibroblasts (Figure 7D). SOD1^G93A^ mouse fibroblasts have a decreased ability to respond to energetic demands in response to stress using glycolytic or oxidative mitochondrial metabolism (Figure 7C).

Because we found changes in the expression of ETC complex proteins in spinal cord, and previous work showed altered mitochondrial function in permeabilized SOD1^G93A^ mouse spinal cord (Cacabelos et al., 2016), we assessed mitochondrial function in end-stage nerve tissue and muscle. Due to technical limitations discussed later, we used the sciatic nerve to analyze mitochondrial function in an affected nerve tissue. We measured simultaneous mitochondrial OCR and hydrogen peroxide and lipid hydroperoxide production rates in permeabilized sciatic nerves and muscle fibers using the OROBOROS Oxygraph-2k with Amplex UltraRed (AUR). Endogenous AUR reaction rate was measured directly after addition of the nerve tissue and muscle fiber bundle to the chamber. OCR and AUR reaction rate were analyzed for both tissues using a sequential addition of glutamate and malate in the absence of ADP to induce proton leak-linked respiration, ADP to stimulate Complex I-linked respiration, pyruvate as a further Complex I substrate, succinate to stimulate Complex I&II-linked respiration (OXPHOS capacity), rotenone to inhibit Complex I and measure Complex II-linked respiration, antimycin A to inhibit Complex III and measure non-mitochondrial OCR, and ascorbate and TMPD to activate CIV-linked respiration. We observe no significant difference in mitochondrial oxidative phosphorylation (OXPHOS) in either sciatic nerve or gastrocnemius muscle (Figure 7E–H). However, there is a significant increase in endogenous AUR reaction rate in gastrocnemius fibers from SOD1^G93A^ mice as well as a trend for an increase in rate in sciatic nerve (Figure 7G,H).

While the probe AUR is generally used as a measurement for production rate of the reactive oxygen species (ROS) hydrogen peroxide, we have previously demonstrated that it also reacts with lipid hydroperoxides produced in muscle in response to loss of innervation (Bhattacharya et al., 2009). Muscles of SOD1^G93A^ mice experience denervation and atrophy and increase expression of genes associated with loss of innervation including acetylcholine receptor subunits (*Chrna1, Chrnd, Chrne*) and *Gadd45a* (Supplemental Figure 2C). *Sln*, which we previously demonstrated is dramatically increased in sarcopenia, is also increased in gastrocnemius muscle from SOD1^G93A^ mice along with the chemokines *Cxcl2* and *Cxcl5* (Qaisar et al., 2018; Supplemental Figure 2C). To distinguish the source of the AUR reaction signal, we treated SOD1^G93A^ gastrocnemius samples with either catalase or arachidonyl trifluoromethyl ketone (AACOCF_3_). AACOCF_3_ is an inhibitor of calcium-dependent phospholipase A2 (cPLA_2_) that we have previously shown inhibits lipid hydroperoxide production in denervated muscle (Bhattacharya et al., 2009). Treatment with AACOCF_3_ more effectively inhibits the endogenous AUR reaction rate than catalase, suggesting the signal is produced primarily by reaction with lipid hydroperoxides and not hydrogen peroxide or superoxide produced by the mitochondrial ETC (Figure 7I). Addition of substrates and inhibitors that increase mitochondrial ETC-derived superoxide and hydrogen peroxide production demonstrates that supraphysiological concentrations of catalase entirely inhibits the increased ETC-derived hydroperoxide production, while AACOCF_3_ only significantly decreases endogenous hydroperoxide production (Supplemental Figures 3A,B).

## Discussion

The goal of this study is to determine the changes in metabolism and stress response in lumbar spinal cord of an ALS mouse model as a function of disease course. Understanding metabolic changes in ALS may help us develop specific dietary interventions that can delay disease progression. We find significant differences in protein content of metabolic pathway enzymes, antioxidant systems, heat shock proteins, and proteases that occur early in the lifespan of the mice and before onset of motor symptoms. Expression of the SOD1^G93A^ mutant protein also leads to tissue specific regulation of chaperones and proteases, specifically, the expression of these proteins is induced in spinal cords but not skeletal muscle from SOD1^G93A^ mice. The differences we observed in metabolism and stress response protein content are explained primarily by expression of the SOD1^G93A^ transgene and not greatly modulated by disease stage.

Several previous studies have utilized proteomics approaches to analyze differences in protein content in the spinal cord of pre-onset ALS rodent models (Lukas et al., 2006; Massignan et al., 2007; Li et al., 2010). Massignan et al. (2007) used the two-dimensional gel electrophoresis proteomic method to analyze protein content changes in whole spinal cords from pre-onset SOD1^G93A^ and control mice and also observed significant changes in content of glycolytic, mitochondrial, and stress response proteins. Li et al. (2010) used both two-dimensional gel electrophoresis as well as liquid chromatography followed by tandem mass spectrometry (MudPIT) to analyze changes in protein content of mitochondria isolated from spinal cords of pre-onset SOD1^G93A^ rats and rats overexpressing wildtype SOD1 (SOD1^WT^). Many proteins identified in their MudPIT analysis were not assessed in our targeted approach. However, Li et al. (2010) also observed many changes pre-onset in metabolic and stress response protein content and some were also observed in our analysis including a decrease in MDH2 and an increase or trend for increase in DECR1, IDH2, and PYGB. Lukas et al. (2006) analyzed protein content of spinal cords from end-stage wildtype, SOD1^WT^, and SOD1^G93A^ fractionated into mitochondrial, soluble, and particulate fractions with two-dimensional liquid chromatography coupled to tandem mass spectrometry. The authors found significant changes in metabolic and antioxidant proteins, among others (Lukas et al., 2006). Previous studies have not analyzed metabolic and stress response protein content as a function of disease course. We report here that modification of metabolic and stress response protein content is primarily due to SOD1^G93A^ expression and not disease course.

### Metabolism Is Altered by SOD1^G93A^ Expression Before Disease Onset

In the early stages of disease development in this ALS mouse model, there is a significant change in the content of proteins related to glycolysis. Transfection of a neuronal cell line to express SOD1^G93A^ protein has been found to increase glycolytic flux (Valbuena et al., 2016). However, at the end stages of the disease, we find that the content of several proteins involved in the malate-aspartate shuttle, pyruvate dehydrogenase, and TCA cycle is decreased, but the content of some proteins related to mitochondrial fatty acid oxidation and the mitochondrial ETC are increased in spinal cords from SOD1^G93A^ mice. Upon activation, astrocytes undergo metabolic changes that optimize glucose utilization to produce lactate including the upregulation of proteins involved in glycolysis (Iglesias et al., 2017). Gliosis in spinal cords from SOD1^G93A^ mice may partially explain the observed increase in some glycolytic proteins. Activated astrocytes also increase beta-oxidation to produce ketone bodies, which is consistent with our finding that HMGCL, a key enzyme in ketogenesis, is upregulated throughout disease course glycolysis (Iglesias et al., 2017). Compared to glial cells, neurons have a higher rate of oxidative metabolism and a slower glycolytic rate (Bélanger et al., 2011). Glucose is shunted through the pentose-phosphate pathway to regenerate NADPH, which is used to reduce glutathione among other reactions in the neuronal antioxidant system (Wamelink et al., 2008). Increased glycolytic rate comes at the expense of the pentose phosphate pathway and cellular antioxidant capacity (Bélanger et al., 2011). The decrease in ECAR also suggests decreased glycolytic potential in SOD1^G93A^ fibroblasts. Altered fatty acid metabolism has been observed in ALS animal models and patients, where hyperlipidemia is associated with increased survival (Schmitt et al., 2014). Interestingly, ACADM catalyzes the first steps of β-oxidation for medium chain fatty acids, and feeding SOD1^G93A^ mice with a medium chain triglyceride-supplemented diet delayed paralysis and motor neuron cell death (Zhao et al., 2012). In addition to fatty acid metabolism, changes in fat mass have also previously been linked to ALS. Decreased body mass index (BMI) is associated with increased risk of ALS (Gallo et al., 2013). Furthermore, high calorie diet appeared to be beneficial in a small phase 2 clinical trial (Wills et al., 2014). Metabolism plays a key role in ALS pathogenesis before onset of symptoms, and therapies targeting metabolism and nutrition have potential to delay disease progression.

Metabolomics experiments have been conducted in ALS patients and mouse models. Metabolomic analysis of skeletal muscle, cerebral cortex, and plasma from wildtype and SOD1^G93A^ mice reveals a distinct metabolite profile in SOD1^G93A^ samples in each tissue (Patin et al., 2016). ALS patients also have significantly different plasma and cerebrospinal fluid (CSF) metabolites compared to controls. Metabolites associated with glucose metabolism are significantly different in the CSF of ALS patients compared to controls (Blasco et al., 2010, 2016; Gray et al., 2015). Significantly different plasma metabolites include amino acids, carbohydrates, lipids, peptides, nucleotides, and xenobiotics (Rozen et al., 2005; Lawton et al., 2012, 2014). The difference in plasma or CSF metabolite levels has been used to predict ALS in patients in preliminary studies, although no definitive metabolite panel has been developed for diagnosis of ALS (Blasco et al., 2014; Lawton et al., 2014). Treatment with Riluzole also significantly alters concentration of several metabolites (Rozen et al., 2005). Metabolite level has also been shown to correlate with disease progression, which suggests that metabolites could be used as a biomarker to diagnose ALS (Kumar et al., 2010; Blasco et al., 2016, 2018). However, there is a significant difference in CSF metabolite profile between sporadic, familial, and familial SOD1 mutant patents (Wuolikainen et al., 2011, 2012). Though metabolite profiles are heterogenous between patient subtypes, metabolite concentrations are clearly altered in ALS patients and future studies may provide evidence of common underlying metabolic pathophysiology.

### Mitochondrial Dysfunction Is Tissue Specific

Mitochondrial dysfunction has been found to occur in numerous studies of ALS tissues, although observed differences are often not consistent between studies. A potential reason for this discrepancy in study outcomes is the diverse genetic causes and disease progressions in patients (Renton et al., 2014). Several studies have shown changes in neuronal ATP levels, ETC complex activities, mitochondrial calcium buffering capacity, and spinal cord astrocyte mitochondrial damage in ALS mouse models (Jung et al., 2002; Ilieva et al., 2009; Cacabelos et al., 2016). Fibroblasts from human patients are commonly studied as a model for mitochondrial function due to their ease of biopsy compared to the primary affected tissues in humans (spinal cord and skeletal muscle). Fibroblast studies in familial and sporadic ALS patient report differences compared to control samples as well as to each other. Familial *TARDBP(p.A328T)* mutant fibroblasts have decreased mitochondrial membrane potential but no change in OCR (Onesto et al., 2016). Familial *C9ORF72* mutant fibroblasts conversely exhibit mitochondrial hyperpolarization, increased OCR and ATP content, increased ROS and increased mitochondrial DNA content (Onesto et al., 2016). Familial SOD1I113T mutants exhibit decreased basal and coupled OCR and decreased reserved capacity (Allen et al., 2014). Sporadic ALS patient fibroblasts presented with decreased basal and coupled OCR (Raman et al., 2015). We observed reduced maximal respiration and reserve capacity in SOD1^G93A^ mutant mouse fibroblasts compared to controls. Together, these findings suggest that ALS pathology affects mitochondrial function in fibroblasts regardless of underlying genetic cause, but mitochondrial phenotypes may be specific to the gene mutation.

ALS is primarily considered a motor neuron disease, and a previous study reported that basal and Complex I-linked respiration were reduced in onset SOD1^G93A^ mouse spinal cords compared to pre-onset controls (Miquel et al., 2014; Cacabelos et al., 2016). However, a major issue with measuring OCR in tissues is that time of oxygen diffusion increases exponentially with distance and the lumbar spinal cord has much larger circumference than the sciatic nerve. Therefore, we chose permeabilized sciatic nerves for nerve tissue measurements, because they have a much smaller radius than spinal cords. Here we report no difference in sciatic nerve tissue OCR but a trend (*p* = 0.059) for an increase in AUR reaction rate. However, protein content data measured in spinal cord demonstrated an increased ETC subunit content from ALS compared to control mice. Sciatic nerve tissue may show similar compensatory changes.

Oxidative phosphorylation has been assayed in permeabilized fibers from ALS patient *M. vastus lateralis* (MVL) muscle biopsy permeabilized fibers finding a reduction in CI-linked OXPHOS (Wiedemann et al., 1998). However, another study of sALS patient MVL permeabilized fibers found no difference between patient and control muscles for CI-, CII-, or CIV-linked respiration (Echaniz-Laguna et al., 2002). The mean duration of disease in this study was low compared to others, and biopsies were taken before most of the patients exhibited MVL muscle atrophy (Echaniz-Laguna et al., 2002). Here we report no significant difference in OCR of permeabilized gastrocnemius fibers from end-stage SOD1^G9EA^ mice compared to control. However, muscle-specific expression of SOD1^G93A^ is sufficient to induce muscle dysmetabolism by inhibiting glucose uptake and shifting muscle toward using lipid metabolism, which suggests muscle metabolism also plays a significant role in ALS hypermetabolism (Dobrowolny et al., 2018).

### Antioxidant and Stress Response Proteins Are Induced

We find that the levels of catalase and peroxiredoxins are induced, but the glutathione system expression is decreased. End-stage sciatic nerve tissue and gastrocnemius muscle fibers from SOD1^G93A^ mice both exhibit increased reactivity *ex vivo* with AUR, which is primarily caused by lipid hydroperoxides in muscle. The role of lipid hydroperoxides in muscle atrophy and contractile dysfunction are still unclear, but these results are consistent with previous findings we reported in denervated muscle fibers (Bhattacharya et al., 2009). GPX4, which reduces lipid hydroperoxides, is not significantly different in spinal cords from SOD1^G93A^ mice. In denervated muscle, we identified cytosolic phospholipase A2 (cPLA_2_) as the primary producer of lipid hydroperoxides (Bhattacharya et al., 2009). Previous studies have shown an upregulation of cPLA_2_ in sporadic ALS patients and pre-onset SOD1^G93A^ mouse spinal cords, and inhibition of cPLA_2_ exhibits protective effects (Solomonov et al., 2016).

Changes in mitochondrial function may play a causal role in ALS pathogenesis for both motor neuron degeneration and skeletal muscle atrophy. Mutant SOD1 localizes to the mitochondrial outer membrane, intermembrane space, and matrix, where it forms protein aggregates possibly including other mitochondrial proteins such as SOD2 (Vijayvergiya et al., 2005). SOD1 mutant protein aggregates form in spinal cord mitochondria before the onset of motor symptoms (Liu et al., 2004), and motor neuron mitochondrial morphology is altered in SOD1 mutants prior to disease onset (Wong et al., 1995; Kong and Xu, 1998). Changes in muscle mitochondrial morphology occur pre-symptomatically, and muscle-specific SOD1^G93A^ expression is sufficient to induce changes in mitochondrial morphology and cause mutant SOD1 aggregation inside mitochondria (Dobrowolny et al., 2008; Luo et al., 2013). We find a significant increase in heat shock proteins and chaperones in spinal cords from SOD1^G93A^ mice throughout disease course. The mitochondrial UPR^mt^ recognizes a decline in mitochondrial protein quality and induces expression of mitochondrial stress response proteins (Hill and Van Remmen, 2014). A previous study found an upregulation of the mitochondrial intermembrane space UPR^mt^ in lumbar spinal cord of SOD1^G93A^ mice (Riar et al., 2017). Here we report mRNA expression of three proteins (*Hspd1, Clpp, Lonp1*) and protein content of 3 proteins (HSPD1, LONP1, CLPX) involved in the mitochondrial matrix UPR^mt^ are increased throughout disease course in spinal cords of SOD1^G93A^ mice (Wu et al., 2014). Despite increased mRNA expression, CLPP protein content is not altered in spinal cords from SOD1^G93A^ mice. CLPP is required in *C. elegans* to initiate the mitochondrial UPR^mt^, though recent evidence suggests it is not required in mammals (Bhaskaran et al., 2018). One hypothesis for the selective vulnerability of motor neurons in ALS is a reduced ability to upregulate stress response proteins in response to metabolic or oxidative stress compared to other neuronal populations (Duan and Mattson, 1999; Batulan et al., 2003; Sinclair, 2005; Mattson et al., 2007). The inability to increase CLPP protein content despite an increase in mRNA expression may provide evidence toward this hypothesis.

Several peroxisomal proteins are differentially regulated in ALS spinal cords. ABCD3 is a peroxisomal ATP-dependent fatty acid transporter with implicated roles in peroxisomal biogenesis (Gärtner and Valle, 1993) and uptake of fatty acid overflow from mitochondrial β-oxidation (van Roermund et al., 2014). Two other proteins involved in peroxisomal quality control are upregulated at end-stage: LONP2 and catalase (CAT). LONP2 is a peroxisomal isoform of the mitochondrial LON protease (LONP1), and both degrade proteins damaged by oxidation (Pomatto et al., 2017). Both LON proteases are increased in SOD1^G93A^ suggesting a response to oxidative damage of proteins in both mitochondria and peroxisomes. SOD1 is expressed in peroxisomes, and peroxisomes are associated with the mitochondrial vacuoles occurring in spinal cords from SOD1^G93A^ mice (Higgins et al., 2003). While wild-type SOD1 has been found in peroxisomes, it is unknown whether SOD1 aggregates form there (Islinger et al., 2009). These results suggest a potential role of peroxisomes in ALS that is poorly understood.

Expression of mitochondrial inner-membrane proteases is also affected (*Afg3l2, Yme1l1, Oma1*). *Yme1l1* and *Oma1* are both inner-membrane proteases involved in mitochondrial fission/fusion dynamics (Quirós et al., 2015). *Afg3l2 and Spg7* are subunits of the mAAA protease, which is responsible for maintaining quality of ETC subunits (Quirós et al., 2015). Alterations in mitochondrial dynamics and morphology have been widely reported in ALS patients and animal models (Muyderman and Chen, 2014). In spinal cords from SOD1^G93A^ mice we observe an increase in protein content of some ETC complex proteins. OPA1 processing by the inner-membrane proteases YME1L1 and OMA1 affects mitochondrial fusion and cristae remodeling (Quirós et al., 2015). YME1L1 cleavage of OPA1 promotes mitochondrial fusion, while OPA1 cleavage by OMA1 is activated by mitochondrial oxidative stress, heat stress, or depolarization and inhibits mitochondrial fusion (Quirós et al., 2015). *Oma1* gene expression is induced in spinal cords from SOD1^G93A^ mice. Increased processing of L-OPA1 by OMA1 is associated with swollen mitochondrial cristae, increased mitochondrial fragmentation, and increased sensitivity to apoptosis (Anand et al., 2014), phenotypes reported in ALS (Muyderman and Chen, 2014). OMA1 activation increases turnover of OMA1 as a feedback loop to limit stress response (Quirós et al., 2015). *Oma1* missense mutations were identified in sporadic ALS patients, however, its role in pathogenesis is unclear (Daoud et al., 2011).

### SOD1^G93A^ Expression Has Tissue Specific Effects

Metabolism and stress response proteins are differentially regulated in spinal cords from SOD1^G93A^ mice and skeletal muscle over the disease course. While UPR^mt^ mRNA and protein content are increased in spinal cords from SOD1^G93A^ mice, we do not observe a similar change in gastrocnemius fibers. Furthermore, mRNA expression of inner mitochondrial membrane proteases is decreased in gastrocnemius muscle. Several previous proteomics studies have been conducted in different tissues or cell lines of ALS models. Engelen-Lee et al. (2017) performed proteomics analysis in spinal cords from post-mortem sporadic ALS patients, and identified 3 differentially expressed proteins in the posterior horn and 18 in the anterior horn. While they observed changes in metabolism and protein folding pathways, no specific proteins were consistent with those reported here in the SOD1^G93A^ familial ALS mouse model. Szelechowski et al. (2018) performed proteomic analysis in cultured primary motor neuron from pre-onset SOD1^G93A^ mice as well as cultured patient fibroblasts. They found significant alterations in metabolic and misfolded protein response pathways in both cell lines. Interestingly, the same malate-aspartate shuttle proteins (GOT1, GOT2, MDH2) we identified as decreased in end-stage spinal cords were all decreased in patient fibroblasts. Capitanio et al. (2012) examined some of the targeted proteins we identified in spinal cords in the gastrocnemius and triceps muscles of pre-onset and onset SOD1^G93A^ mice. Only ALDH2 and CRYAB are consistently differentially regulated between spinal cord and muscle samples for stress response. αB-crystallin (CRYAB) has been extensively studied as a potential treatment for ALS treatment, because over-expression reduced SOD1 aggregates in cell culture models and knockout in SOD1 mutant mice accelerated disease onset (Xu et al., 2015). However, transgenic overexpression of αB-crystallin in SOD1 mutant mouse lines did not slow disease progression or reduce aggregate formation, making it a poor therapeutic target for ALS (Xu et al., 2015). The only significant differences in mitochondrial metabolism enzymes conserved between muscle and spinal cord are UQCRC1 and ATP5B, which are both increased in onset triceps and spinal cord (Capitanio et al., 2012). Elf et al. (2014) performed proteomics analysis in muscles of control and ALS patients. In ALS patients, ACADM, PGAM, and PRDX6 (*p* < 0.06) were also found to be differentially regulated. Few changes in ALS protein content compared to control appear to be conserved between tissues.

### Caveats and Future Directions

This study provides a view of metabolism in the spinal cord of an ALS model throughout disease course. However, it is impossible to separate metabolism of different cell types in the spinal cord using this design. A recent proteomics study on cultured primary motor neurons from SOD1^G93A^ mice found upregulation of oxidative metabolism including fatty acid β-oxidation and TCA cycle and downregulation of glycolysis (Szelechowski et al., 2018). In addition, survival of motor neurons from an ALS mouse model depended on fatty acid β-oxidation to a much greater extent than neurons from wild-type mice (Szelechowski et al., 2018). Combined, these data suggest these metabolic changes are occurring *in vivo* in motor neurons before onset of motor symptoms. Thus, metabolic reprogramming may play a role in early ALS pathogenesis and provides rationale for further development of dietary interventions as well as other interventions targeting metabolism in ALS patients.

This study also provides evidence for upregulation of components of the mitochondrial UPR^mt^ in spinal cords of SOD1^G93A^ mice before onset of symptoms. Whether upregulation of this pathway delays onset of symptoms requires further study and could provide a new avenue for development of ALS treatments.

## Materials and Methods

### Animals

All animal experiments were carried out in accordance with protocol approved by Institutional Animal Care and Use Committee at Oklahoma Medical Research Foundation. The G93A^SOD1^ mice [B6-Tg(SOD1-G93A)1Gur/J(G93AGur1)] were purchased from Jackson Laboratories (stock number 004435, Jackson Laboratories, Bar Harbor, Maine) and were bred on a C57BL/6J background from our lab and a colony was established for use in this study. The colony is updated with mice purchased from Jackson laboratories every 10–12 months to maintain the stability of the colony. Mice were caged in a pathogen free environment with free access to standard chow and water and maintained on a 12 h light/dark cycle. Mice were weighed weekly and disease assessment was carried out as described earlier (Evans et al., 2015). Based on the severity of the disease, male and female mice were classified as pre-onset (63–69 days), onset (102–121 days), and end stage (135–152 days) and sacrificed accordingly. Wild-type littermates of same age served as controls. Mice were monitored and euthanized if symptoms became too severe. At the time of sacrifice, brain, spinal cord, sciatic nerve, epigonadal white adipose tissue (eWAT), quadriceps, soleus, and gastrocnemius were dissected, weighed and flash frozen in liquid nitrogen for biochemical analysis. One batch of mice was sacrificed separately and fresh sciatic nerve was used for the O2K measurements.

### Quantification of Protein Content Using Targeted Proteomics

Protein was isolated from lumbar mouse spinal cords of male and female wild-type and SOD1^G93A^ (pre-onset, onset, and end-stage, *n* = 5–6) in RIPA buffer: 10 mM Tris-Cl (pH 8.0), 1 mM EDTA, 1% Triton X-100 (v/v), 0.1% sodium deoxycholate (w/v), 0.1% SDS (w/v), 140 mM NaCL, and 1 mM PMSF, with protease inhibitor cocktail (Calbiochem Set III, EDTA-free; EMD Millipore; Billerica, MA, United States) as previously described (Ahn et al., 2018). An aliquot containing 100 μg of each samples was taken, 8 pmol BSA was added as an internal standard, and the samples were incubated at 80°C for 15 min in 1% SDS (w/v). Proteins were precipitated with 80% acetone (v/v) at -20°C overnight. The dried protein pellet was reconstituted in 100 μl Laemmli sample buffer and run into a short (1.5 cm) SDS-PAGE gel. The gels were fixed and stained. Each sample was cut from the gel as the entire lane and divided into smaller pieces. The gel pieces were washed to remove the Coomassie blue then reduced, alkylated, and digested overnight with trypsin. The mixture of peptides was extracted from the gel, evaporated to dryness in a SpeedVac and reconstituted in 150 μl 1% acetic acid (v/v) for analysis.

The analyses were carried out on a TSQ Vantage triple quadrupole mass spectrometry system (ThermoScientific TSQ Vantage, San Jose, CA, United States). The HPLC was an Eksigent splitless nanoflow system with a 10 cm × 75 μm i.d. C18 reversed phase capillary column (Eksigent, Dublin, CA, United States). 7 μl aliquots were injected and the peptide eluted with a 60 min gradient of acetonitrile in 0.1% formic acid (v/v). The mass spectrometer was operated in the selected reaction monitoring mode. For each protein, the method was developed to measure 2 ideal peptides. Assays for multiple proteins were bundled together in larger panels. Data were analyzed using the program SkyLine to determine the integrated peak area of the appropriate chromatographic peaks. The response for each protein was calculated as the geometric mean of the two peptide area. These values were normalized to the response for the BSA standard and multiple housekeeping proteins were also monitored. Entrez Gene name and ID and UniprotKB entry, name, and gene names, and protein names are listed in Supplemental Table 1 along with results for each protein. The Skyline files containing peak identification and analysis is available at: https://zenodo.org/record/2633276 (doi: 10.5281/zenodo.2633276).

### Quantitative Real-Time Polymerase Chain Reaction (RT-PCR)

RT-PCR was performed as previously described (Sataranatarajan et al., 2015). Total RNA was extracted from gastrocnemius and lumbar spinal cord of male and female mice using TRIzol reagent (Invitrogen, Carlsbad, CA, United States). Equal amounts of extracted RNA (1 μg) were converted to first strand cDNA using a cDNA synthesis kit (Bio-Rad, Herculus, CA, United States). 5 ng of the cDNA samples was amplified using primers for *Afg3l2, Cxcl2, Cxcl5, Chrna1, Chrnd, Chrne, Clpp, Gadd45a, Hspd1, Lonp1, mt-Atp6, mt-Co2, mt-Nd1, Ndufs3, Oma1, Ppargc1a, Rfesd, Rn18s* (18S rRNA), *Sdha, Sdhb, Sln, Spg7*, and *Yme1l1* (details in Supplemental Table 2) and SYBER green (Invitrogen, Carlsbad, CA, United States). Real time PCR (RT-PCR) was done in Quant Studio 6 (Applied Biosystems, Foster City, CA, United States). The ΔΔC_t_ method was used to calculate relative mRNA expression.

### Primary Fibroblast Cell Culture

Primary fibroblasts were isolated from tail snips of wild-type and SOD1^G93A^ mice as previously described (Pharaoh et al., 2016). Briefly, snips were washed, minced, and incubated overnight with DMEM supplemented with Liberase DL (Roche). The next day the samples were suspended in complete DMEM (4.5 g/l glucose DMEM supplemented with 10% Fetal Bovine Serum and 1% Penicillin/Streptomycin), centrifuged at 200 × *g* for 5 min, and the cell pellet was resuspended in complete DMEM and transferred to a 25 mL cell culture flask.

### Mitochondrial Function

Oxygen consumption rate (OCR) and extracellular acidification rate (ECAR) of dermal fibroblasts (30,000 cells/well, *n* = 5, three independent experiments) was measured with a Seahorse XF24 Flux Analyzer under basal conditions and after sequential addition of 1 μM oligomycin (Complex V inhibitor), 1 μM FCCP (proton uncoupler), and 1 μM antimycin A (Complex III inhibitor) normalized to total protein (μg per well) measured by Bradford Protein Assay with BSA standard as previously described (Pharaoh et al., 2016). All OCR measurements were corrected for non-mitochondrial respiration (NMR) using the antimycin A values; basal is the third baseline value, maximal is the highest FCCP value corrected for NMR, reserve capacity is the difference between basal and maximal respiration, ATP-linked respiration is the difference between basal and the lowest oligomycin value, and proton leak is the lowest oligomycin value. For ECAR, basal is the third baseline value, and stressed is the highest value recorded after addition of oligomycin and FCCP.

Gastrocnemius fibers and sciatic nerves were collected from end-stage female mice and placed in ice-cold buffer X for mitochondrial function measurements. OCR and the rate of hydrogen peroxide and lipid hydroperoxide production were simultaneously measured using the Oxygraph-2k (O2k, OROBOROS Instruments, Innsbruck, Austria) in gastrocnemius fibers as previously reported with minor modifications (Ahn et al., 2018). We mechanically separated ∼3–5 mg of red gastrocnemius fibers with forceps in buffer X, permeabilized their plasma membrane with saponin (30 μg/ml) for 30 min, and washed them (3 × 5 min) in buffer Z at 4°C. We mechanically separated sciatic nerves nerve and minced them into 1–1.5 mm segments in buffer X, permeabilized their plasma membrane with saponin (50 μg/ml) for 30min, and washed them (3 × 5 min) in buffer Z at 4°C (Zheng et al., 2011).

OCR was measured using an oxygen probe, while AUR reaction rate was measured using the O2k-Fluo LED2-Module Fluorescence-Sensor Green. Measurements were performed on permeabilized fibers and sciatic nerve sections in buffer Z media at 37°C containing 10 μM Amplex^®^ UltraRed (Molecular Probes, Eugene, OR), 1 U/ml horseradish peroxidase (HRP), 2.5 U/ml superoxide dismutase 1 (SOD1), and blebbistatin (25 μM). HRP catalyzes the reaction between peroxides, including hydrogen peroxide and lipid hydroperoxides, and AUR to produce the fluorescent resorufin (excitation: 565 nm; emission: 600 nM). The fluorescent signal was normalized between chambers via a hydrogen peroxide standard curve established on each day of experiments. Rates of respiration and hydrogen peroxide and lipid hydroperoxide production were measured during sequential additions of substrates and inhibitors as follows: baseline, glutamate (10 mM) and malate (2 mM) for Leak, ADP (5 mM) for Complex I-linked respiration, pyruvate (5 mM) for maximal Complex I-linked respiration, succinate (10 mM) for Complex I and II-linked respiration, rotenone (1μM) for Complex II-linked respiration, antimycin A (1 μM) to measure NMR, and TMPD (0.5 mM) immediately followed by ascorbate (5 mM) for Complex IV-linked respiration. All respiration measurements were normalized to antimycin A to account for non-mitochondrial oxygen consumption. Background resorufin production was subtracted from each experimental measurement of hydrogen peroxide and lipid hydroperoxide production. Graphs for AUR reaction rate show measurements from baseline, after addition of rotenone, and after addition of antimycin A. Data for both OCR and rates of hydrogen peroxide and lipid hydroperoxide generation were normalized by milligrams of muscle bundle wet weights. All substrates and inhibitors were prepared according to the protocols from OROBOROS instruments.

For the catalase vs. AACOCF_3_ study, 2000 U/ml of catalase was added during permeabilization, washes, and assay, while 20 μM AACOCF_3_ was added during permeabilization and washes. 2000 U/ml catalase was used as a final concentration for experimentation in order to have maximal inhibition. One unit of catalase will decompose 1.0 μmole of hydrogen peroxide per minute. Because our chambers are 2 ml, and we use 2000 U/ml, our catalase concentration is capable of decomposing 4 mmoles hydrogen peroxide H_2_O_2_ per minute. Our measurements are in the pmole hydrogen peroxide per minute scale.

### Statistical Analyses and Data Visualization

Statistical analysis and visualization were performed using GraphPad Prism version 7.0b for OS X (GraphPad Software, La Jolla, CA, United States^1^). For RT-PCR and targeted proteomics data sets, outliers were removed using the Grubbs’ outlier test (Alpha = 0.05). Statistical significance for protein content was determined by ordinary two-way ANOVA with Tukey’s Multiple Comparison test and Benjamini–Hochberg procedure. “Genotype” denotes a significant genotype effect in two-way ANOVA with *q* < 0.05 but no individual Tukey Multiple Comparison test was significant. Significance symbols demonstrate *q* < 0.05 for a genotype effect and additional significant results for the Tukey Multiple Comparison test. Values for CAT, GPI1, HSPD1, and MDH1 were the average from multiple experiments.

Statistical significance for RT-PCR and longitudinal tissue masses is determined by ordinary two-way ANOVA with Tukey’s Multiple Comparison test. “Genotype” denotes a significant genotype effect in Benjamini-Hochberg corrected two-way ANOVA with *p* < 0.05 but no individual Multiple Comparison test was significant. Significance symbols demonstrate *p* < 0.05 for a genotype effect and additional significant results for the Tukey Multiple Comparison test. For eWAT end-stage mass, statistical significance is determined by unpaired two-tailed student’s *t*-test (^∗^*p* < 0.05). Unpaired two-tailed student’s *t*-test was used for comparisons between two groups in the mitochondrial function experiments, and ordinary one-way ANOVA with Tukey’s Multiple Comparison Test was used for the OROBOROS O2K experiment of gastrocnemius fibers from SOD1^G93A^ mice treated with catalase or AACOCF_3_.

Heatmap and the PCA plot were generated using ClustVis with default settings (Row scaling = unit variance scaling, PCA method = SVD with imputation, clustering distance for rows = correlation, clustering method for rows = average, tree ordering for rows = tightest cluster first) (Metsalu and Vilo, 2015).

## Ethics Statement

This study was carried out in accordance with the recommendations of USDA guidelines by the Institutional Animal Care and Use Committee at Oklahoma Medical Research Foundation. The protocol was approved by the Institutional Animal Care and Use Committee at Oklahoma Medical Research Foundation.

## Author Contributions

Study was conceived and designed by HVR. Experiments were performed and data collected by GP, KSa, KSt, SH, JG, CK, and MK. BA developed the sciatic nerve mitochondrial experimental procedure used here. Data analysis and visualization was performed by GP. The manuscript was written by GP with input from HVR. All authors edited and approved the final manuscript.

## Conflict of Interest Statement

The authors declare that the research was conducted in the absence of any commercial or financial relationships that could be construed as a potential conflict of interest.

## Footnotes

1 www.graphpad.com
