# Supplementary figures and images for: Metabolic and Stress Response Changes Precede Disease Onset in the Spinal Cord of Mutant SOD1 ALS Mice
Source: Front Neurosci. 2019 May 31;13:487. doi: 10.3389/fnins.2019.00487 (PMC6554287; doi:10.3389/fnins.2019.00487)

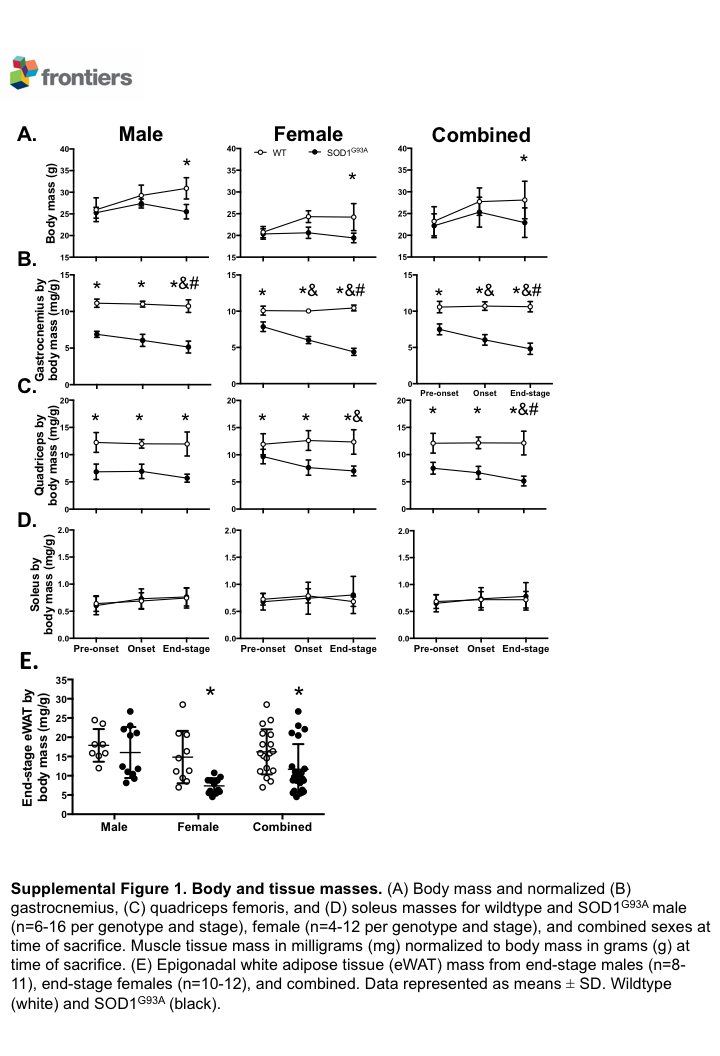

Supplement: Supplementary file 1 [file Image_1.TIFF]

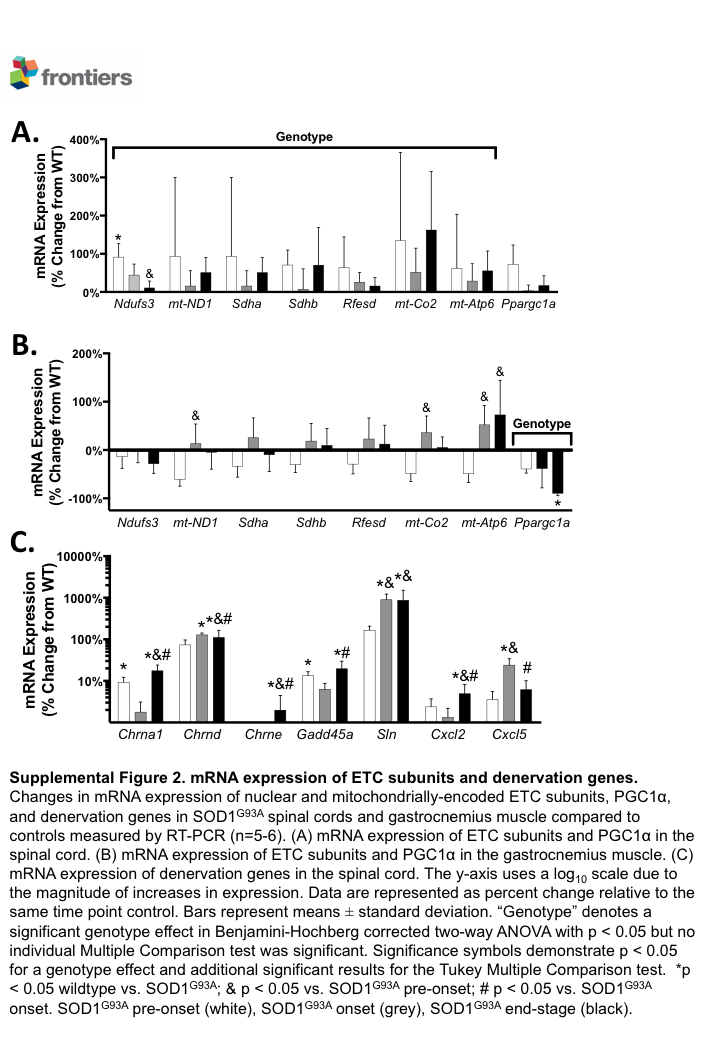

Supplement: Supplementary file 2 [file Image_2.TIFF]

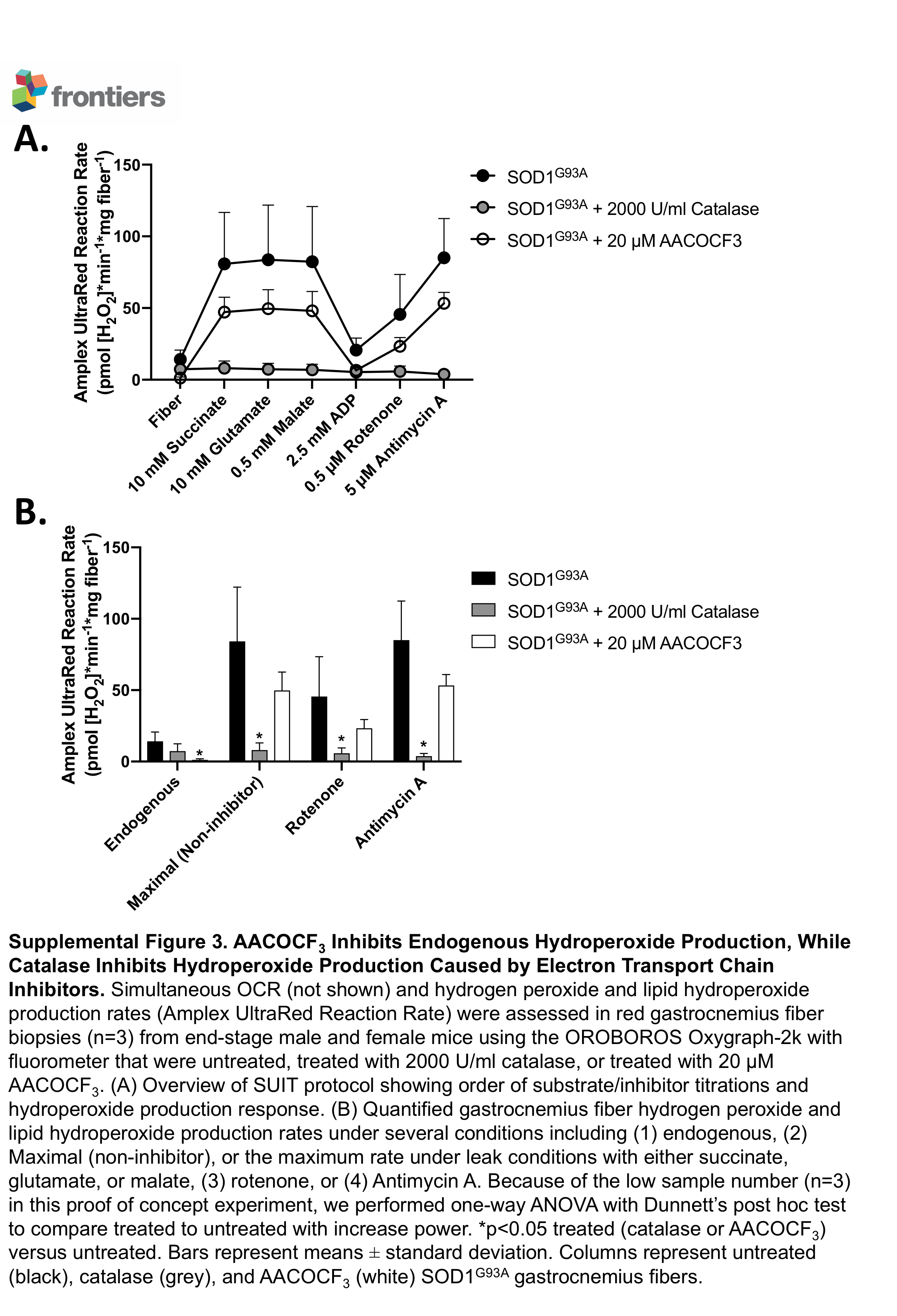

Supplement: Supplementary file 3 [file Image_3.TIFF]
