# Supplementary material for: Metabolic and Stress Response Changes Precede Disease Onset in the Spinal Cord of Mutant SOD1 ALS Mice
Source: Front Neurosci. 2019 May 31;13:487. doi: 10.3389/fnins.2019.00487 (PMC6554287; doi:10.3389/fnins.2019.00487)
Supplement: Supplementary file 4 [file Table_1.DOCX]

Supplementary Material

Metabolic and Stress Response Changes Precede Disease Onset in the Spinal Cord of Mutant SOD1 ALS Mice

**Gavin Pharaoh, Kavithalakshmi Sataranatarajan, Kaitlyn Riddle, Shauna Hill, Jake Gregston, Bumsoo Ahn, Caroline Kinter, Michael Kinter, and Holly Van Remmen***

*** Correspondence:** Holly Van Remmen: [Holly-VanRemmen@omrf.org](mailto:Holly-VanRemmen@omrf.org)

# Supplementary Figures and Tables

**Supplemental Table 1. Targeted proteomics data.** A list of all gene names, Entrez gene ID, and protein names identified in the Selected Reaction Monitoring proteomics experiment in spinal cords from wildtype and SOD1^G93A^ mice [(Maglott et al., 2011)](https://paperpile.com/c/tT8dSY/qwwj). ND = not detected. ACOX1, ALDOB, CD36, CPT1B, ENO3, FABP1, HMGCS2, J23PDK4, NNT, PECR, PHB, PHB2, PRDX4, PRKACA, SAMM50, SLC25A4, SLC2A4, TALDO1, and TUFM did not reach the limit of detection in any samples.

| **Entrez Gene Name** | **Entrez Gene ID** | **UniProtKB Entry** | **UniprotKB Entry name** | **UniProtKB Gene names** |  | **WT** | | | | | | **SOD1G93A** | | | | | |
| --- | --- | --- | --- | --- | --- | --- | --- | --- | --- | --- | --- | --- | --- | --- | --- | --- | --- |
|  |  |  |  |  |  | **Pre-Onset** | | **Onset** | | **End-stage** | | **Pre-Onset** | | **Onset** | | **End-stage** | |
|  |  |  |  |  | **Protein Name** | **Average ± SD** | | **Average ± SD** | | **Average ± SD** | | **Average ± SD** | | **Average ± SD** | | **Average ± SD** | |
| Abcd3 | 19299 | P55096 | ABCD3_MOUSE | Abcd3 Pmp70 Pxmp1 | ATP-binding cassette, sub-family D (ALD), member 3 | 0.114 | 0.013 | 0.105 | 0.013 | 0.101 | 0.009 | 0.123 | 0.018 | 0.130 | 0.020 | 0.146 | 0.010 |
| Acaa1a | 113868 | Q921H8 | THIKA_MOUSE | Acaa1a Acaa1 | acetyl-Coenzyme A acyltransferase 1A | 0.072 | 0.009 | 0.084 | 0.027 | 0.080 | 0.011 | 0.070 | 0.014 | 0.066 | 0.021 | 0.074 | 0.016 |
| Acaa1b | 235674 | Q8VCH0 | THIKB_MOUSE | Acaa1b Acaa1 | acetyl-Coenzyme A acyltransferase 1B |  |  |  |  |  |  |  |  |  |  |  |  |
| Acaa2 | 52538 | Q8BWT1 | THIM_MOUSE | Acaa2 | acetyl-Coenzyme A acyltransferase 2 (mitochondrial 3-oxoacyl-Coenzyme A thiolase) | 0.201 | 0.051 | 0.173 | 0.025 | 0.208 | 0.036 | 0.179 | 0.041 | 0.170 | 0.013 | 0.195 | 0.041 |
| Acad11 | 102632 | Q80XL6 | ACD11_MOUSE | Acad11 | acyl-Coenzyme A dehydrogenase family, member 11 | 0.014 | 0.001 | 0.013 | 0.003 | 0.014 | 0.003 | 0.015 | 0.005 | 0.014 | 0.002 | 0.016 | 0.002 |
| Acadl | 11363 | P51174 | ACADL_MOUSE | Acadl | acyl-Coenzyme A dehydrogenase, long-chain | 0.180 | 0.024 | 0.196 | 0.052 | 0.219 | 0.032 | 0.208 | 0.044 | 0.220 | 0.042 | 0.232 | 0.046 |
| Acadm | 11364 | P45952 | ACADM_MOUSE | Acadm | acyl-Coenzyme A dehydrogenase, medium chain | 0.095 | 0.019 | 0.073 | 0.007 | 0.085 | 0.007 | 0.105 | 0.012 | 0.114 | 0.014 | 0.121 | 0.003 |
| Acads | 11409 | Q07417 | ACADS_MOUSE | Acads | acyl-Coenzyme A dehydrogenase, short chain | 0.038 | 0.005 | 0.032 | 0.003 | 0.041 | 0.005 | 0.039 | 0.008 | 0.038 | 0.005 | 0.043 | 0.010 |
| Acadvl | 11370 | P50544 | ACADV_MOUSE | Acadvl Vlcad | acyl-Coenzyme A dehydrogenase, very long chain | 0.090 | 0.024 | 0.075 | 0.008 | 0.089 | 0.015 | 0.093 | 0.017 | 0.088 | 0.010 | 0.095 | 0.009 |
| Aco2 | 11429 | Q99KI0 | ACON_MOUSE | Aco2 | aconitase 2, mitochondrial | 3.710 | 0.630 | 3.477 | 0.748 | 4.199 | 0.362 | 3.472 | 0.610 | 3.270 | 0.224 | 3.345 | 0.413 |
| Acot13 | 66834 | Q9CQR4 | ACO13_MOUSE | Acot13 Them2 | acyl-CoA thioesterase 13 | 0.738 | 0.100 | 0.679 | 0.142 | 0.748 | 0.068 | 0.588 | 0.114 | 0.537 | 0.087 | 0.536 | 0.093 |
| Acsl1 | 14081 | P41216 | ACSL1_MOUSE | Acsl1 Acsl2 Facl2 | acyl-CoA synthetase long-chain family member 1 | 0.073 | 0.015 | 0.061 | 0.009 | 0.076 | 0.013 | 0.080 | 0.015 | 0.077 | 0.004 | 0.068 | 0.008 |
| Akr1b1 | 11677 | P45376 | ALDR_MOUSE | Akr1b1 Akr1b3 Aldor1 Aldr1 | aldo-keto reductase family 1, member B3 (aldose reductase) | 0.251 | 0.023 | 0.280 | 0.046 | 0.344 | 0.058 | 0.262 | 0.070 | 0.303 | 0.097 | 0.262 | 0.048 |
| Aldh2 | 11669 | P47738 | ALDH2_MOUSE | Aldh2 Ahd-1 Ahd1 | aldehyde dehydrogenase 2, mitochondrial | 0.437 | 0.080 | 0.387 | 0.033 | 0.503 | 0.031 | 0.544 | 0.103 | 0.578 | 0.055 | 0.562 | 0.050 |
| Aldoa | 11674 | P05064 | ALDOA_MOUSE | Aldoa Aldo1 | aldolase A, fructose-bisphosphate | 3.546 | 0.650 | 3.620 | 0.162 | 4.469 | 1.157 | 3.342 | 0.931 | 3.104 | 0.861 | 2.815 | 0.555 |
| Atp2a2 | 11938 | O55143 | AT2A2_MOUSE | Atp2a2 | ATPase, Ca++ transporting, cardiac muscle, slow twitch 2 | 0.359 | 0.046 | 0.317 | 0.035 | 0.362 | 0.043 | 0.319 | 0.034 | 0.284 | 0.026 | 0.291 | 0.025 |
| Atp5a1 | 11946 | Q03265 | ATPA_MOUSE | Atp5f1a Atp5a1 | ATP synthase, H+ transporting, mitochondrial F1 complex, alpha subunit 1 | 7.643 | 0.978 | 7.005 | 0.227 | 7.330 | 0.860 | 10.854 | 0.883 | 11.505 | 1.200 | 11.328 | 1.523 |
| Atp5b | 11947 | P56480 | ATPB_MOUSE | Atp5f1b Atp5b | ATP synthase, H+ transporting mitochondrial F1 complex, beta subunit | 4.553 | 0.588 | 4.504 | 0.157 | 4.372 | 0.713 | 10.761 | 0.930 | 11.363 | 0.893 | 10.716 | 1.276 |
| Bdh1 | 71911 | Q80XN0 | BDH_MOUSE | Bdh1 Bdh | 3-hydroxybutyrate dehydrogenase, type 1 | 1.142 | 0.151 | 0.742 | 0.067 | 0.763 | 0.067 | 1.126 | 0.038 | 0.911 | 0.149 | 0.859 | 0.124 |
| Calr | 12317 | P14211 | CALR_MOUSE | Calr | calreticulin | 1.246 | 0.235 | 1.016 | 0.130 | 1.145 | 0.171 | 1.277 | 0.082 | 1.195 | 0.163 | 1.364 | 0.155 |
| Cat | 12359 | P24270 | CATA_MOUSE | Cat Cas-1 Cas1 | catalase | 0.177 | 0.020 | 0.151 | 0.019 | 0.148 | 0.021 | 0.219 | 0.041 | 0.192 | 0.043 | 0.208 | 0.037 |
| Clpp | 53895 | O88696 | CLPP_MOUSE | Clpp | caseinolytic mitochondrial matrix peptidase proteolytic subunit | 0.373 | 0.050 | 0.323 | 0.071 | 0.384 | 0.054 | 0.348 | 0.070 | 0.307 | 0.037 | 0.332 | 0.063 |
| Clpx | 270166 | Q9JHS4 | CLPX_MOUSE | Clpx | caseinolytic mitochondrial matrix peptidase chaperone subunit | ND |  | ND |  | ND |  | 0.037 | 0.009 | 0.036 | 0.007 | 0.032 | 0.002 |
| Coq6 | 217707 | Q8R1S0 | COQ6_MOUSE | Coq6 | coenzyme Q6 homolog (yeast) | 0.029 | 0.006 | 0.028 | 0.008 | 0.027 | 0.007 | 0.040 | 0.011 | 0.039 | 0.006 | 0.042 | 0.012 |
| Cpt1a | 12894 | P97742 | CPT1A_MOUSE | Cpt1a Cpt-1 Cpt1 | carnitine palmitoyltransferase 1a, liver | 0.146 | 0.015 | 0.132 | 0.007 | 0.136 | 0.003 | 0.147 | 0.034 | 0.142 | 0.019 | 0.156 | 0.020 |
| Cpt2 | 12896 | P52825 | CPT2_MOUSE | Cpt2 Cpt-2 | carnitine palmitoyltransferase 2 | 0.041 | 0.008 | 0.038 | 0.007 | 0.044 | 0.005 | 0.048 | 0.009 | 0.044 | 0.006 | 0.053 | 0.005 |
| Crat | 12908 | P47934 | CACP_MOUSE | Crat | carnitine acetyltransferase | 0.041 | 0.006 | 0.033 | 0.003 | 0.038 | 0.003 | 0.041 | 0.009 | 0.037 | 0.005 | 0.041 | 0.006 |
| Crot | 74114 | Q9DC50 | OCTC_MOUSE | Crot Cot | carnitine O-octanoyltransferase | 0.009 | 0.003 | 0.013 | 0.003 | 0.011 | 0.003 | 0.014 | 0.004 | 0.015 | 0.004 | 0.016 | 0.003 |
| Cryab | 12955 | P23927 | CRYAB_MOUSE | Cryab Crya2 | crystallin, alpha B | 0.082 | 0.013 | 0.087 | 0.014 | 0.088 | 0.015 | 0.114 | 0.003 | 0.146 | 0.036 | 0.153 | 0.030 |
| Cs | 12974 | Q9CZU6 | CISY_MOUSE | Cs | citrate synthase | 4.169 | 0.638 | 3.823 | 0.504 | 4.659 | 0.476 | 4.086 | 0.819 | 3.923 | 0.688 | 3.650 | 0.662 |

| **Entrez Gene Name** | **Entrez Gene ID** | **UniProtKB Entry** | **UniprotKB Entry name** | **UniProtKB Gene names** |  | **WT** | | | | | | **SOD1G93A** | | | | | |
| --- | --- | --- | --- | --- | --- | --- | --- | --- | --- | --- | --- | --- | --- | --- | --- | --- | --- |
|  |  |  |  |  |  | **Pre-Onset** | | **Onset** | | **End-stage** | | **Pre-Onset** | | **Onset** | | **End-stage** | |
|  |  |  |  |  | **Protein Name** | **Average ± SD** | | **Average ± SD** | | **Average ± SD** | | **Average ± SD** | | **Average ± SD** | | **Average ± SD** | |
| Decr1 | 67460 | Q9CQ62 | DECR_MOUSE | Decr1 | 2,4-dienoyl CoA reductase 1, mitochondrial | 0.199 | 0.035 | 0.177 | 0.009 | 0.215 | 0.041 | 0.220 | 0.043 | 0.247 | 0.027 | 0.267 | 0.034 |
| Dlat | 235339 | Q8BMF4 | ODP2_MOUSE | Dlat | dihydrolipoamide S-acetyltransferase (E2 component of pyruvate dehydrogenase complex) | 3.509 | 0.633 | 3.142 | 0.093 | 4.115 | 0.507 | 2.608 | 0.305 | 2.387 | 0.454 | 1.591 | 1.198 |
| Dld | 13382 | O08749 | DLDH_MOUSE | Dld | dihydrolipoamide dehydrogenase | 0.372 | 0.063 | 0.335 | 0.041 | 0.385 | 0.088 | 0.817 | 0.189 | 0.760 | 0.119 | 0.837 | 0.155 |
| Dlst | 78920 | Q9D2G2 | ODO2_MOUSE | Dlst | dihydrolipoamide S-succinyltransferase (E2 component of 2-oxo-glutarate complex) | 2.008 | 0.247 | 1.705 | 0.023 | 2.138 | 0.329 | 2.454 | 0.315 | 2.476 | 0.311 | 2.361 | 0.301 |
| Ech1 | 51798 | O35459 | ECH1_MOUSE | Ech1 | enoyl coenzyme A hydratase 1, peroxisomal | 0.134 | 0.020 | 0.107 | 0.009 | 0.133 | 0.016 | 0.135 | 0.029 | 0.141 | 0.014 | 0.158 | 0.005 |
| Echs1 | 93747 | Q8BH95 | ECHM_MOUSE | Echs1 | enoyl Coenzyme A hydratase, short chain, 1, mitochondrial | 0.244 | 0.048 | 0.215 | 0.034 | 0.257 | 0.019 | 0.250 | 0.062 | 0.238 | 0.046 | 0.255 | 0.031 |
| Eci1 | 13177 | P42125 | ECI1_MOUSE | Eci1 Dci | enoyl-Coenzyme A delta isomerase 1 | 0.115 | 0.029 | 0.108 | 0.018 | 0.139 | 0.018 | 0.101 | 0.006 | 0.107 | 0.019 | 0.121 | 0.020 |
| Eci2 | 23986 | Q9WUR2 | ECI2_MOUSE | Eci2 Peci | enoyl-Coenzyme A delta isomerase 2 | 0.053 | 0.011 | 0.044 | 0.007 | 0.053 | 0.008 | 0.054 | 0.009 | 0.050 | 0.010 | 0.056 | 0.012 |
| Ehhadh | 74147 | Q9DBM2 | ECHP_MOUSE | Ehhadh | enoyl-Coenzyme A, hydratase/3-hydroxyacyl Coenzyme A dehydrogenase | 1.958 | 0.187 | 1.329 | 0.643 | 1.258 | 0.565 | 0.614 | 0.091 | 0.603 | 0.063 | 0.591 | 0.066 |
| Eno1 | 13806 | P17182 | ENOA_MOUSE | Eno1 Eno-1 | enolase 1, alpha non-neuron | 5.529 | 0.531 | 5.234 | 0.895 | 6.092 | 0.801 | 5.353 | 1.012 | 5.677 | 0.861 | 4.636 | 1.126 |
| Etfa | 110842 | Q99LC5 | ETFA_MOUSE | Etfa | electron transferring flavoprotein, alpha polypeptide | 0.440 | 0.081 | 0.439 | 0.111 | 0.521 | 0.071 | 0.414 | 0.095 | 0.463 | 0.070 | 0.416 | 0.052 |
| Etfb | 110826 | Q9DCW4 | ETFB_MOUSE | Etfb | electron transferring flavoprotein, beta polypeptide | 0.359 | 0.066 | 0.348 | 0.033 | 0.401 | 0.071 | 0.428 | 0.111 | 0.444 | 0.037 | 0.479 | 0.061 |
| Etfdh | 66841 | Q921G7 | ETFD_MOUSE | Etfdh | electron transferring flavoprotein, dehydrogenase | 0.185 | 0.032 | 0.166 | 0.030 | 0.199 | 0.028 | 0.198 | 0.036 | 0.176 | 0.007 | 0.191 | 0.019 |
| Fabp3 | 14077 | P11404 | FABPH_MOUSE | Fabp3 Fabph1 | fatty acid binding protein 3, muscle and heart | 0.115 | 0.025 | 0.107 | 0.033 | 0.085 | 0.018 | 0.186 | 0.051 | 0.161 | 0.024 | 0.149 | 0.039 |
| Fabp4 | 11770 | P04117 | FABP4_MOUSE | Fabp4 Ap2 | fatty acid binding protein 4, adipocyte | 0.019 | 0.007 | 0.034 | 0.027 | 0.018 | 0.004 | 0.020 | 0.004 | 0.024 | 0.015 | 0.030 | 0.014 |
| Fh1 | 14194 | P97807 | FUMH_MOUSE | Fh Fh1 | fumarate hydratase 1 | 0.544 | 0.112 | 0.520 | 0.111 | 0.629 | 0.102 | 0.522 | 0.112 | 0.548 | 0.089 | 0.450 | 0.101 |
| Gapdh | 14433 | P16858 | G3P_MOUSE | Gapdh Gapd | glyceraldehyde-3-phosphate dehydrogenase | 38.248 | 4.600 | 36.015 | 3.442 | 41.843 | 7.118 | 33.987 | 7.227 | 30.545 | 4.309 | 27.554 | 7.933 |
| Glud1 | 14661 | P26443 | DHE3_MOUSE | Glud1 Glud | glutamate dehydrogenase 1 | 3.804 | 0.681 | 3.018 | 0.114 | 3.847 | 0.637 | 4.699 | 0.825 | 4.526 | 0.613 | 4.536 | 0.668 |
| Got1 | 14718 | P05201 | AATC_MOUSE | Got1 | glutamic-oxaloacetic transaminase 1, soluble | 2.926 | 0.420 | 2.692 | 0.363 | 3.606 | 0.564 | 2.601 | 0.409 | 2.352 | 0.421 | 2.366 | 0.597 |
| Got2 | 14719 | P05202 | AATM_MOUSE | Got2 Got-2 | glutamatic-oxaloacetic transaminase 2, mitochondrial | 4.369 | 0.869 | 4.272 | 1.020 | 5.084 | 0.637 | 3.394 | 0.554 | 3.170 | 0.665 | 3.019 | 0.684 |
| Gpi1 | 14751 | P06745 | G6PI_MOUSE | Gpi Gpi1 | glucose phosphate isomerase 1 | 0.693 | 0.090 | 0.693 | 0.110 | 0.803 | 0.108 | 0.946 | 0.126 | 1.014 | 0.130 | 1.017 | 0.181 |
| Gpx1 | 14775 | P11352 | GPX1_MOUSE | Gpx1 | glutathione peroxidase 1 | 0.092 | 0.026 | 0.082 | 0.027 | 0.096 | 0.014 | 0.052 | 0.008 | 0.067 | 0.011 | 0.064 | 0.028 |
| Gpx4 | 625249 | O70325 | GPX4_MOUSE | Gpx4 | glutathione peroxidase 4 | 0.307 | 0.057 | 0.291 | 0.048 | 0.362 | 0.039 | 0.327 | 0.031 | 0.327 | 0.052 | 0.306 | 0.052 |
| Gsr | 14782 | P47791 | GSHR_MOUSE | Gsr Gr1 | glutathione reductase | 0.127 | 0.035 | 0.121 | 0.020 | 0.169 | 0.025 | 0.145 | 0.025 | 0.142 | 0.023 | 0.149 | 0.024 |
| Gsta3 | 14859 | P30115 | GSTA3_MOUSE | Gsta3 Gstyc | glutathione S-transferase, alpha 3 | 0.357 | 0.090 | 0.338 | 0.069 | 0.411 | 0.114 | 0.130 | 0.030 | 0.115 | 0.015 | 0.122 | 0.028 |
| Gstm1 | 14862 | P10649 | GSTM1_MOUSE | Gstm1 | glutathione S-transferase, mu 1 | 2.703 | 0.424 | 2.601 | 0.381 | 2.913 | 0.161 | 2.342 | 0.366 | 2.157 | 0.128 | 2.698 | 0.526 |
| Gstp1 | 14870 | P19157 | GSTP1_MOUSE | Gstp1 Gstpib | glutathione S-transferase, pi 1 | 2.054 | 0.135 | 1.811 | 0.267 | 2.110 | 0.295 | 1.907 | 0.418 | 1.712 | 0.133 | 1.888 | 0.319 |
| Hadh | 15107 | Q61425 | HCDH_MOUSE | Hadh Hadhsc Mschad Schad | hydroxyacyl-Coenzyme A dehydrogenase | 0.175 | 0.027 | 0.143 | 0.017 | 0.169 | 0.020 | 0.288 | 0.060 | 0.303 | 0.027 | 0.317 | 0.058 |
| Hadha | 97212 | Q8BMS1 | ECHA_MOUSE | Hadha | hydroxyacyl-Coenzyme A dehydrogenase/3-ketoacyl-Coenzyme A thiolase/enoyl-Coenzyme A hydratase (trifunctional protein), alpha subunit | 0.520 | 0.097 | 0.423 | 0.048 | 0.523 | 0.065 | 0.530 | 0.091 | 0.506 | 0.056 | 0.548 | 0.074 |
| Hadhb | 231086 | Q99JY0 | ECHB_MOUSE | Hadhb | hydroxyacyl-Coenzyme A dehydrogenase/3-ketoacyl-Coenzyme A thiolase/enoyl-Coenzyme A hydratase (trifunctional protein), beta subunit | 0.551 | 0.109 | 0.472 | 0.072 | 0.534 | 0.063 | 0.535 | 0.079 | 0.567 | 0.039 | 0.523 | 0.115 |
| Hk1 | 15275 | P17710 | HXK1_MOUSE | Hk1 | hexokinase 1 | 0.704 | 0.048 | 0.534 | 0.076 | 0.615 | 0.122 | 0.808 | 0.141 | 0.871 | 0.079 | 0.824 | 0.091 |
| Hmgcl | 15356 | P38060 | HMGCL_MOUSE | Hmgcl | 3-hydroxy-3-methylglutaryl-Coenzyme A lyase | 0.125 | 0.021 | 0.102 | 0.009 | 0.121 | 0.010 | 0.153 | 0.023 | 0.123 | 0.028 | 0.139 | 0.017 |
| Hmgcs1 | 208715 | Q8JZK9 | HMCS1_MOUSE | Hmgcs1 | 3-hydroxy-3-methylglutaryl-Coenzyme A synthase 1 | 0.348 | 0.056 | 0.225 | 0.035 | 0.258 | 0.031 | 0.312 | 0.033 | 0.188 | 0.028 | 0.141 | 0.038 |
| Hsd17b4 | 15488 | P51660 | DHB4_MOUSE | Hsd17b4 Edh17b4 | hydroxysteroid (17-beta) dehydrogenase 4 | 0.270 | 0.024 | 0.193 | 0.008 | 0.255 | 0.032 | 0.279 | 0.045 | 0.261 | 0.028 | 0.302 | 0.055 |
| Hsp90b1 | 22027 | P08113 | ENPL_MOUSE | Hsp90b1 Grp94 Tra-1 Tra1 | heat shock protein 90, beta (Grp94), member 1 | 0.680 | 0.082 | 0.625 | 0.159 | 0.747 | 0.102 | 1.027 | 0.164 | 0.947 | 0.206 | 1.020 | 0.181 |
| Hspa1a | 193740 | Q61696 | HS71A_MOUSE | Hspa1a Hsp70-3 Hsp70A1 | heat shock protein 1A | 5.812 | 0.947 | 5.308 | 0.511 | 6.030 | 1.169 | 6.753 | 0.906 | 6.421 | 1.606 | 5.730 | 0.516 |
| Hspa5 | 14828 | P20029 | BIP_MOUSE | Hspa5 Grp78 | heat shock protein 5 | 0.519 | 0.114 | 0.394 | 0.061 | 0.457 | 0.089 | 0.406 | 0.045 | 0.457 | 0.119 | 0.431 | 0.072 |
| Hspa9 | 15526 | P38647 | GRP75_MOUSE | Hspa9 Grp75 Hsp74 Hspa9a | heat shock protein 9 | 0.651 | 0.087 | 0.596 | 0.088 | 0.727 | 0.057 | 0.730 | 0.134 | 0.709 | 0.068 | 0.623 | 0.070 |
| Hspd1 | 15510 | P63038 | CH60_MOUSE | Hspd1 Hsp60 | heat shock protein 1 (chaperonin) | 1.548 | 0.183 | 1.428 | 0.151 | 1.584 | 0.177 | 2.089 | 0.292 | 1.943 | 0.428 | 1.926 | 0.307 |
| Idh1 | 15926 | O88844 | IDHC_MOUSE | Idh1 | isocitrate dehydrogenase 1 (NADP+), soluble | 0.688 | 0.066 | 0.513 | 0.032 | 0.648 | 0.100 | 0.675 | 0.076 | 0.590 | 0.066 | 0.551 | 0.127 |
| Idh2 | 269951 | P54071 | IDHP_MOUSE | Idh2 | isocitrate dehydrogenase 2 (NADP+), mitochondrial | 0.236 | 0.042 | 0.206 | 0.030 | 0.266 | 0.048 | 0.255 | 0.051 | 0.274 | 0.032 | 0.305 | 0.040 |
| Idh3a | 67834 | Q9D6R2 | IDH3A_MOUSE | Idh3a | isocitrate dehydrogenase 3 (NAD+) alpha | 1.985 | 0.355 | 1.673 | 0.197 | 2.177 | 0.288 | 1.614 | 0.203 | 1.430 | 0.232 | 1.371 | 0.291 |
| Idh3b | 170718 | Q91VA7 | Q91VA7_MOUSE | Idh3b mCG_9915 | isocitrate dehydrogenase 3 (NAD+) beta | 1.036 | 0.059 | 0.990 | 0.074 | 1.230 | 0.159 | 1.119 | 0.184 | 0.980 | 0.157 | 1.022 | 0.181 |
| Idh3g | 15929 | P70404 | IDHG1_MOUSE | Idh3g | isocitrate dehydrogenase 3 (NAD+), gamma | 1.446 | 0.258 | 1.339 | 0.293 | 1.616 | 0.162 | 1.567 | 0.282 | 1.416 | 0.269 | 1.372 | 0.216 |
| Ldha | 16828 | P06151 | LDHA_MOUSE | Ldha Ldh-1 Ldh1 | lactate dehydrogenase A | 0.919 | 0.230 | 0.747 | 0.046 | 1.125 | 0.269 | 1.033 | 0.295 | 1.106 | 0.227 | 1.092 | 0.234 |
| Ldhb | 16832 | P16125 | LDHB_MOUSE | Ldhb Ldh-2 Ldh2 | lactate dehydrogenase B | 4.532 | 0.772 | 4.628 | 0.525 | 5.868 | 0.828 | 4.989 | 1.080 | 4.385 | 0.494 | 4.055 | 1.191 |
| Lonp1 | 74142 | Q8CGK3 | LONM_MOUSE | Lonp1 Prss15 | lon peptidase 1, mitochondrial | 0.122 | 0.020 | 0.117 | 0.020 | 0.148 | 0.030 | 0.185 | 0.034 | 0.173 | 0.019 | 0.168 | 0.023 |
| Lonp2 | 66887 | Q9DBN5 | LONP2_MOUSE | Lonp2 | lon peptidase 2, peroxisomal | 0.007 | 0.002 | 0.007 | 0.003 | 0.006 | 0.001 | 0.007 | 0.001 | 0.007 | 0.002 | 0.010 | 0.002 |
| Mdh1 | 17449 | P14152 | MDHC_MOUSE | Mdh1 Mor2 | malate dehydrogenase 1, NAD (soluble) | 5.567 | 0.756 | 5.549 | 0.517 | 6.629 | 0.544 | 6.799 | 0.981 | 6.687 | 0.649 | 5.781 | 1.385 |

| **Entrez Gene Name** | **Entrez Gene ID** | **UniProtKB Entry** | **UniprotKB Entry name** | **UniProtKB Gene names** |  | **WT** | | | | | | **SOD1G93A** | | | | | |
| --- | --- | --- | --- | --- | --- | --- | --- | --- | --- | --- | --- | --- | --- | --- | --- | --- | --- |
|  |  |  |  |  |  | **Pre-Onset** | | **Onset** | | **End-stage** | | **Pre-Onset** | | **Onset** | | **End-stage** | |
|  |  |  |  |  | **Protein Name** | **Average ± SD** | | **Average ± SD** | | **Average ± SD** | | **Average ± SD** | | **Average ± SD** | | **Average ± SD** | |
| Mdh2 | 17448 | P08249 | MDHM_MOUSE | Mdh2 Mor1 | malate dehydrogenase 2, NAD (mitochondrial) | 5.794 | 1.112 | 5.262 | 0.303 | 6.386 | 0.873 | 4.692 | 1.334 | 4.554 | 0.904 | 3.978 | 0.833 |
| Msra | 110265 | Q9D6Y7 | MSRA_MOUSE | Msra | methionine sulfoxide reductase A | 0.102 | 0.003 | 0.113 | 0.019 | 0.118 | 0.017 | 0.113 | 0.019 | 0.098 | 0.006 | 0.098 | 0.008 |
| Ndufs1 | 227197 | Q91VD9 | NDUS1_MOUSE | Ndufs1 | NADH dehydrogenase (ubiquinone) Fe-S protein 1 | 0.550 | 0.121 | 0.510 | 0.117 | 0.486 | 0.089 | 0.563 | 0.111 | 0.707 | 0.156 | 0.592 | 0.062 |
| Ndufv1 | 17995 | Q91YT0 | NDUV1_MOUSE | Ndufv1 | NADH dehydrogenase (ubiquinone) flavoprotein 1 | 0.326 | 0.072 | 0.310 | 0.094 | 0.299 | 0.061 | 0.394 | 0.077 | 0.482 | 0.117 | 0.396 | 0.059 |
| Ogdh | 18293 | Q60597 | ODO1_MOUSE | Ogdh Kiaa4192 | oxoglutarate (alpha-ketoglutarate) dehydrogenase (lipoamide) | 1.095 | 0.196 | 0.950 | 0.167 | 1.203 | 0.150 | 1.199 | 0.252 | 1.086 | 0.105 | 1.092 | 0.166 |
| Pcx | 18563 | Q05920 | PYC_MOUSE | Pc Pcx | pyruvate carboxylase | 0.538 | 0.085 | 0.495 | 0.056 | 0.599 | 0.132 | 0.628 | 0.145 | 0.541 | 0.035 | 0.541 | 0.089 |
| Pdha1 | 18597 | P35486 | ODPA_MOUSE | Pdha1 Pdha-1 | pyruvate dehydrogenase E1 alpha 1 | 2.265 | 0.327 | 2.190 | 0.170 | 2.747 | 0.322 | 2.133 | 0.384 | 2.049 | 0.322 | 1.896 | 0.377 |
| Pdhb | 68263 | Q9D051 | ODPB_MOUSE | Pdhb | pyruvate dehydrogenase (lipoamide) beta | 1.571 | 0.252 | 1.440 | 0.244 | 1.736 | 0.211 | 1.360 | 0.203 | 1.320 | 0.207 | 1.191 | 0.236 |
| Pfkl | 18641 | P12382 | PFKAL_MOUSE | Pfkl Pfk-l Pfkb | phosphofructokinase, liver, B-type | 0.306 | 0.055 | 0.326 | 0.036 | 0.395 | 0.074 | 0.341 | 0.023 | 0.366 | 0.072 | 0.405 | 0.047 |
| Pfkm | 18642 | P47857 | PFKAM_MOUSE | Pfkm Pfk-m Pfka | phosphofructokinase, muscle | 0.746 | 0.204 | 0.827 | 0.145 | 0.891 | 0.295 | 0.983 | 0.249 | 0.868 | 0.241 | 0.957 | 0.229 |
| Pgam2 | 56012 | O70250 | PGAM2_MOUSE | Pgam2 | phosphoglycerate mutase 2 | ND |  | ND |  | ND |  | 0.017 | 0.007 | 0.029 | 0.009 | 0.031 | 0.018 |
| Pgk1 | 18655 | P09411 | PGK1_MOUSE | Pgk1 Pgk-1 | phosphoglycerate kinase 1 | 2.015 | 0.374 | 2.024 | 0.297 | 2.267 | 0.096 | 1.640 | 0.319 | 1.793 | 0.465 | 1.713 | 0.360 |
| Pkm2 | 18746 | P52480 | KPYM_MOUSE | Pkm Pk3 Pkm2 Pykm | pyruvate kinase | 9.773 | 1.642 | 10.263 | 1.439 | 12.072 | 2.545 | 11.193 | 1.446 | 11.041 | 1.172 | 10.243 | 1.692 |
| Prdx1 | 18477 | P35700 | PRDX1_MOUSE | Prdx1 Msp23 Paga Tdpx2 | peroxiredoxin 1 | 1.311 | 0.165 | 1.170 | 0.267 | 1.251 | 0.128 | 1.396 | 0.172 | 1.294 | 0.148 | 1.480 | 0.223 |
| Prdx2 | 21672 | Q61171 | PRDX2_MOUSE | Prdx2 Tdpx1 Tpx | peroxiredoxin 2 | 0.619 | 0.095 | 0.596 | 0.083 | 0.639 | 0.145 | 0.746 | 0.114 | 0.781 | 0.095 | 0.688 | 0.039 |
| Prdx3 | 11757 | P20108 | PRDX3_MOUSE | Prdx3 Aop1 Mer5 | peroxiredoxin 3 | 0.402 | 0.069 | 0.316 | 0.080 | 0.409 | 0.043 | 0.497 | 0.081 | 0.503 | 0.069 | 0.489 | 0.073 |
| Prdx5 | 54683 | P99029 | PRDX5_MOUSE | Prdx5 Prdx6 | peroxiredoxin 5 | 1.271 | 0.163 | 1.055 | 0.169 | 1.276 | 0.259 | 1.457 | 0.222 | 1.183 | 0.188 | 1.049 | 0.164 |
| Prdx6 | 11758 | O08709 | PRDX6_MOUSE | Prdx6 Aop2 Ltw4 Prdx5 | peroxiredoxin 6 | 1.254 | 0.125 | 1.231 | 0.279 | 1.370 | 0.152 | 1.087 | 0.150 | 1.223 | 0.191 | 1.858 | 0.332 |
| Pygb | 110078 | Q8CI94 | PYGB_MOUSE | Pygb | brain glycogen phosphorylase | 1.149 | 0.187 | 0.908 | 0.034 | 1.242 | 0.217 | 1.255 | 0.263 | 1.209 | 0.205 | 1.146 | 0.155 |
| Pygm | 19309 | Q9WUB3 | PYGM_MOUSE | Pygm | muscle glycogen phosphorylase | 0.596 | 0.146 | 0.495 | 0.093 | 0.694 | 0.117 | 0.697 | 0.118 | 0.626 | 0.066 | 0.583 | 0.081 |
| Sdha | 66945 | Q8K2B3 | SDHA_MOUSE | Sdha | succinate dehydrogenase complex, subunit A, flavoprotein (Fp) | 0.796 | 0.098 | 0.725 | 0.042 | 0.819 | 0.063 | 0.831 | 0.094 | 0.795 | 0.061 | 0.772 | 0.088 |
| Sdhb | 67680 | Q9CQA3 | SDHB_MOUSE | Sdhb | succinate dehydrogenase complex, subunit B, iron sulfur (Ip) | 0.523 | 0.058 | 0.465 | 0.043 | 0.527 | 0.082 | 0.446 | 0.098 | 0.419 | 0.064 | 0.432 | 0.061 |
| Sdhc | 66052 | Q9CZB0 | C560_MOUSE | Sdhc | succinate dehydrogenase complex, subunit C, integral membrane protein | 0.094 | 0.012 | 0.068 | 0.011 | 0.080 | 0.017 | 0.090 | 0.020 | 0.080 | 0.003 | 0.079 | 0.009 |
| Slc25a11 | 67863 | Q9CR62 | M2OM_MOUSE | Slc25a11 | solute carrier family 25 (mitochondrial carrier oxoglutarate carrier), member 11 | 0.878 | 0.060 | 0.728 | 0.119 | 0.764 | 0.157 | 0.848 | 0.128 | 0.993 | 0.171 | 0.948 | 0.077 |
| Slc25a20 | 57279 | Q9Z2Z6 | MCAT_MOUSE | Slc25a20 Cac Cact | solute carrier family 25 (mitochondrial carnitine/acylcarnitine translocase), member 20 | 0.093 | 0.026 | 0.095 | 0.006 | 0.074 | 0.018 | 0.072 | 0.015 | 0.091 | 0.027 | 0.082 | 0.015 |
| Sod1 | 20655 | P08228 | SODC_MOUSE | Sod1 | superoxide dismutase 1, soluble | 1.177 | 0.210 | 1.137 | 0.266 | 1.172 | 0.206 | 3.734 | 0.773 | 3.948 | 0.895 | 3.916 | 0.530 |
| Sod2 | 20656 | P09671 | SODM_MOUSE | Sod2 Sod-2 | superoxide dismutase 2, mitochondrial | 0.984 | 0.139 | 0.795 | 0.115 | 1.046 | 0.203 | 1.033 | 0.304 | 0.863 | 0.043 | 0.833 | 0.137 |
| Sucla2 | 20916 | Q9Z2I9 | SUCB1_MOUSE | Sucla2 | succinate-Coenzyme A ligase, ADP-forming, beta subunit | 1.449 | 0.254 | 1.318 | 0.051 | 1.737 | 0.212 | 1.548 | 0.187 | 1.527 | 0.238 | 1.418 | 0.276 |
| Suclg1 | 56451 | Q9WUM5 | SUCA_MOUSE | Suclg1 | succinate-CoA ligase, GDP-forming, alpha subunit | 0.476 | 0.092 | 0.469 | 0.115 | 0.520 | 0.069 | 0.470 | 0.070 | 0.441 | 0.061 | 0.400 | 0.080 |
| Tkt | 21881 | P40142 | TKT_MOUSE | Tkt | transketolase | 1.199 | 0.194 | 1.222 | 0.184 | 1.292 | 0.292 | 1.417 | 0.160 | 1.491 | 0.398 | 1.528 | 0.254 |
| Tpi1 | 21991 | P17751 | TPIS_MOUSE | Tpi1 Tpi | triosephosphate isomerase 1 | 1.990 | 0.257 | 1.581 | 0.344 | 2.280 | 0.456 | 1.953 | 0.061 | 1.650 | 0.187 | 1.759 | 0.307 |
| Txn1 | 22166 | P10639 | THIO_MOUSE | Txn Txn1 | thioredoxin 1 | 0.505 | 0.087 | 0.508 | 0.107 | 0.507 | 0.136 | 0.523 | 0.097 | 0.453 | 0.084 | 0.451 | 0.088 |
| Txnrd1 | 50493 | Q9JMH6 | TRXR1_MOUSE | Txnrd1 Trxr1 | thioredoxin reductase 1 | 0.169 | 0.012 | 0.184 | 0.031 | 0.213 | 0.024 | 0.205 | 0.019 | 0.220 | 0.040 | 0.200 | 0.031 |
| Uqcrc1 | 22273 | Q9CZ13 | QCR1_MOUSE | Uqcrc1 | ubiquinol-cytochrome c reductase core protein 1 | 1.473 | 0.262 | 1.485 | 0.221 | 1.320 | 0.258 | 1.949 | 0.332 | 2.225 | 0.319 | 2.016 | 0.256 |
