# Supplementary material for: Metabolic and Stress Response Changes Precede Disease Onset in the Spinal Cord of Mutant SOD1 ALS Mice
Source: Front Neurosci. 2019 May 31;13:487. doi: 10.3389/fnins.2019.00487 (PMC6554287; doi:10.3389/fnins.2019.00487)
Supplement: Supplementary file 5 [file Table_2.DOCX]

Supplementary Material

Metabolic and Stress Response Changes Precede Disease Onset in the Spinal Cord of Mutant SOD1 ALS Mice

**Gavin Pharaoh, Kavithalakshmi Sataranatarajan, Kaitlyn Riddle, Shauna Hill, Jake Gregston, Bumsoo Ahn, Caroline Kinter, Michael Kinter, and Holly Van Remmen***

*** Correspondence:** Holly Van Remmen: [Holly-VanRemmen@omrf.org](mailto:Holly-VanRemmen@omrf.org)

# Supplementary Figures and Tables

**Supplemental Table 2. RT-PCR information.** RT-PCR gene name, gene ID, and primer sets.

| **RT-PCR Primer Sequences** | | | |
| --- | --- | --- | --- |
| **Gene Name** | **NCBI Gene ID** | **Forward** | **Reverse** |
| *Afg3l2* | 69597 | TCTCTGCTGAGGGATGTAATTGC | TCCTTTGGGAGGTCGAGAACA |
| *Cxcl2* | 20310 | GAGCTTGAGTGTGACGCCCCCAGG | GTTAGCCTTGCCTTTGTTCAGTATC |
| *Cxcl5* | 20311 | GCATTTCTGTTGCTGTTCACGCTG | CCTCCTTCTGGTTTTTCAGTTTAGC |
| *Chrna1* | 11435 | ACCTGGACCTATGACGGCTCT | AGTTACTCAGGTCGGGCTGGT |
| *Chrnd* | 11447 | CATCGAGTGGATCATCATTGAC | CGGCGGATGATAAGGTAGAA |
| *Chrne* | 11448 | GATTGGCATTGACTGGCACG | CCACTCCAAACTGCCCATC |
| *Clpp* | 53895 | GCCTTGCCGTGCATTTCTC | CTCCACCACTATGGGGATGA |
| *Gadd45a* | 13197 | AGACCGAAAGGATGGACACG | GTACACGCCGACCGTAATG |
| *Hspd1* | 15510 | CACAGTCCTTCGCCAGATGAG | CTACACCTTGAAGCATTAAGGCT |
| *Lonp1* | 74142 | AGGATCTTGCCTTGTGTGGA | TGGATGAGGAGCTGAGCAAG |
| *mt-Atp6* | 17705 | ACACACCAAAAGGACGAACA | GAAGGAAGTGGGCAAGTGAG |
| *mt-Co2* | 17709 | ATGGCCTACCCATTCCAACT | CGGGGTTGTTGATTTCGTC |
| *mt-Nd1* | 17716 | AATCGCCATAGCCTTCCTAAC | TGGTATTGGTAGGGGAACTCA |
| *Ndufs3* | 68349 | CTGTGGCAGCACGTAAGAAG | ACTCATCAAGGCAGGACACC |
| *Oma1* | 67013 | TCTCTGGAGTGAATAACCTGGC | GCACTTGAGAGGCATCTTGATT |
| *Ppargc1a* | 19017 | CAGACCTGACACAACGCGGACAG | CCTGCGCAAGCTTCTCTGAGC |
| *Rfesd* | 218341 | TGGTCTCCCAGTTTGTTTCC | GCAGCTTCCTGGTCAATCTC |
| *Rn18s* | 19791 | GTGGAGCGATTTGTCTGGTT | CGCTGAGCCAGTCAGTGTAG |
| *Sdha* | 66945 | CAGAAGTCGATGCAGAACCA | CGACCCGCACTTTGTAATCT |
| *Sdhb* | 67680 | GGAGGGCAAGCAACAGTATC | GCGTTCCTCTGTGAAGTCGT |
| *Sln* | 66402 | GAGGTGGAGAGACTGAGGTCCTTGG | GAAGCTCGGGGCACACAGCAG |
| *Spg7* | 234847 | TTGCTATTGCGAATACTGACCC | CTGCTTCATCCTTGAGGTGTT |
| *Yme1l1* | 27377 | GCCTCAGGTTACAATTCCTCTC | CACAGGCTCACTACTGGGA |
