# Supplementary material for: Metabolic and Stress Response Changes Precede Disease Onset in the Spinal Cord of Mutant SOD1 ALS Mice
Source: Front Neurosci. 2019 May 31;13:487. doi: 10.3389/fnins.2019.00487 (PMC6554287; doi:10.3389/fnins.2019.00487)
Supplement: Supplementary file 6 [file Table_3.DOCX]

Supplementary Material

Metabolic and Stress Response Changes Precede Disease Onset in the Spinal Cord of Mutant SOD1 ALS Mice

**Gavin Pharaoh, Kavithalakshmi Sataranatarajan, Kaitlyn Riddle, Shauna Hill, Jake Gregston, Bumsoo Ahn, Caroline Kinter, Michael Kinter, and Holly Van Remmen***

*** Correspondence:** Holly Van Remmen: [Holly-VanRemmen@omrf.org](mailto:Holly-VanRemmen@omrf.org)

# Supplementary Figures and Tables

**Supplemental Table 3. Spinal cord RT-PCR data.** RT-PCR relative quantification (RQ) values from spinal cords of wildtype and SOD1^G93A^ mice at all disease stages**.** *p < 0.05 wildtype vs. SOD1^G93A^; & p < 0.05 vs. SOD1^G93A^ pre-onset; # p < 0.05 vs. SOD1^G93A^ onset.

|  | **Spinal Cord (RQ ± standard deviation)** | | | | | | | | | | | | | | | | | |  |
| --- | --- | --- | --- | --- | --- | --- | --- | --- | --- | --- | --- | --- | --- | --- | --- | --- | --- | --- | --- |
|  | **WT** | | | | | | | | | **SOD1^G93A^** | | | | | | | | | **Genotype Effect** |
| **Gene ID** | **Pre-onset** | | | **Onset** | | | **End-stage** | | | **Pre-onset** | | | **Onset** | | | **End-stage** | | |  |
| *Afg3l2* | 0.88 | ± | 0.20 | 1.01 | ± | 0.16 | 1.02 | ± | 0.21 | 1.39 | ± | 0.31* | 1.06 | ± | 0.22 | 1.39 | ± | 0.31 | * |
| *Clpp* | 1.04 | ± | 0.31 | 1.06 | ± | 0.40 | 1.12 | ± | 0.56 | 2.34 | ± | 0.39* | 1.15 | ± | 0.61& | 1.50 | ± | 0.84 | * |
| *Hspd1* | 1.02 | ± | 0.24 | 1.03 | ± | 0.27 | 1.16 | ± | 0.61 | 1.49 | ± | 0.21 | 1.27 | ± | 0.82 | 1.71 | ± | 1.00 | * |
| *Lonp1* | 1.07 | ± | 0.42 | 1.06 | ± | 0.43 | 1.13 | ± | 0.60 | 2.12 | ± | 0.31* | 1.44 | ± | 0.83 | 1.66 | ± | 0.70 | * |
| *mt-Atp6* | 0.83 | ± | 0.55 | 1.09 | ± | 0.52 | 1.03 | ± | 0.25 | 1.34 | ± | 1.17 | 1.41 | ± | 0.49 | 1.60 | ± | 0.53 | * |
| *mt-Co2* | 0.82 | ± | 0.48 | 1.26 | ± | 0.77 | 1.02 | ± | 0.25 | 1.94 | ± | 1.89 | 1.92 | ± | 0.78 | 2.69 | ± | 1.56 | * |
| *mt-Nd1* | 0.80 | ± | 0.41 | 1.09 | ± | 0.52 | 1.02 | ± | 0.22 | 1.55 | ± | 1.65 | 1.27 | ± | 0.43 | 1.55 | ± | 0.41 | p=0.07 |
| *Ndufs3* | 1.07 | ± | 0.41 | 1.04 | ± | 0.33 | 1.06 | ± | 0.38 | 2.05 | ± | 0.37* | 1.51 | ± | 0.30 | 1.18 | ± | 0.19& | * |
| *Oma1* | 1.04 | ± | 0.29 | 1.12 | ± | 0.61 | 1.09 | ± | 0.50 | 1.96 | ± | 0.23* | 2.42 | ± | 0.43* | 1.73 | ± | 0.27 | * |
| *Ppargc1a* | 0.84 | ± | 0.30 | 1.02 | ± | 0.26 | 1.73 | ± | 1.24 | 1.46 | ± | 0.42 | 1.06 | ± | 0.15 | 2.04 | ± | 0.44 | NS |
| *Rfesd* | 1.05 | ± | 0.37 | 1.07 | ± | 0.44 | 1.03 | ± | 0.25 | 1.72 | ± | 0.84 | 1.35 | ± | 0.26 | 1.19 | ± | 0.22 | * |
| *Sdha* | 1.07 | ± | 0.43 | 1.06 | ± | 0.40 | 1.12 | ± | 0.61 | 1.89 | ± | 0.83 | 1.31 | ± | 0.68 | 1.84 | ± | 0.89 | * |
| *Sdhb* | 1.04 | ± | 0.30 | 1.02 | ± | 0.22 | 0.89 | ± | 0.13 | 1.78 | ± | 0.40 | 1.09 | ± | 0.54 | 1.52 | ± | 0.88 | * |
| *Spg7* | 0.78 | ± | 0.33 | 1.02 | ± | 0.23 | 0.91 | ± | 0.10 | 1.16 | ± | 0.33 | 1.07 | ± | 0.22 | 0.95 | ± | 0.19 | p=0.09 |
| *Yme1l1* | 0.85 | ± | 0.19 | 1.03 | ± | 0.25 | 1.03 | ± | 0.26 | 1.34 | ± | 0.39 | 1.34 | ± | 0.25 | 0.83 | ± | 0.22&# | * |
