# Supplementary material for: Metabolic and Stress Response Changes Precede Disease Onset in the Spinal Cord of Mutant SOD1 ALS Mice
Source: Front Neurosci. 2019 May 31;13:487. doi: 10.3389/fnins.2019.00487 (PMC6554287; doi:10.3389/fnins.2019.00487)
Supplement: Supplementary file 7 [file Table_4.DOCX]

Supplementary Material

Metabolic and Stress Response Changes Precede Disease Onset in the Spinal Cord of Mutant SOD1 ALS Mice

**Gavin Pharaoh, Kavithalakshmi Sataranatarajan, Kaitlyn Riddle, Shauna Hill, Jake Gregston, Bumsoo Ahn, Caroline Kinter, Michael Kinter, and Holly Van Remmen***

*** Correspondence:** Holly Van Remmen: [Holly-VanRemmen@omrf.org](mailto:Holly-VanRemmen@omrf.org)

# Supplementary Figures and Tables

**Supplemental Table 4. Gastrocnemius RT-PCR data.** RT-PCR relative quantification (RQ) values from gastrocnemius muscles of wildtype and SOD1^G93A^ mice at all disease stages**.** *p < 0.05 wildtype vs. SOD1^G93A^; & p < 0.05 vs. SOD1^G93A^ pre-onset; # p < 0.05 vs. SOD1^G93A^ onset.

|  | **Gastrocnemius (RQ ± standard deviation)** | | | | | | | | | | | | | | | | | |  |
| --- | --- | --- | --- | --- | --- | --- | --- | --- | --- | --- | --- | --- | --- | --- | --- | --- | --- | --- | --- |
|  | **Wildtype** | | | | | | | | | **SOD1^G93A^** | | | | | | | | | **Genotype Effect** |
| **Gene ID** | **Pre-onset** | | | **Onset** | | | **End-stage** | | | **Pre-onset** | | | **Onset** | | | **End-stage** | | |  |
| *Afg3l2* | 0.84 | ± | 0.20 | 0.85 | ± | 0.39 | 1.68 | ± | 1.28 | 0.98 | ± | 0.16 | 0.72 | ± | 0.39 | 0.66 | ± | 0.32 | NS |
| *Cxcl2* | 0.76 | ± | 0.20 | 1.18 | ± | 0.60 | 1.21 | ± | 0.75 | 2.58 | ± | 0.98 | 2.78 | ± | 1.00 | 7.24 | ± | 3.94*&# | * |
| *Cxcl5* | 0.64 | ± | 0.32 | 1.31 | ± | 1.04 | 1.05 | ± | 0.34 | 2.93 | ± | 1.26 | 32.93 | ± | 13.33*& | 7.58 | ± | 4.08# | * |
| *Chrna1* | 0.90 | ± | 0.79 | 1.96 | ± | 2.17 | 0.92 | ± | 0.59 | 9.32 | ± | 2.66* | 5.48 | ± | 2.56 | 17.21 | ± | 5.89*&# | * |
| *Chrnd* | 0.81 | ± | 0.29 | 0.86 | ± | 0.13 | 1.67 | ± | 1.28 | 61.03 | ± | 18.01 | 112.73 | ± | 10.71* | 189.56 | ± | 88.74*&# | * |
| *Chrne* | 1.00 | ± | 0.10 | 1.02 | ± | 0.22 | 1.75 | ± | 0.28 | 0.93 | ± | 0.26 | 1.49 | ± | 0.33 | 5.28 | ± | 4.34*&# | * |
| *Clpp* | 1.03 | ± | 0.28 | 1.09 | ± | 0.47 | 1.07 | ± | 0.45 | 0.76 | ± | 0.07 | 1.08 | ± | 0.13 | 0.72 | ± | 0.26 | NS |
| *Gadd45a* | 0.99 | ± | 0.91 | 0.98 | ± | 0.82 | 1.00 | ± | 0.59 | 14.42 | ± | 3.03* | 7.25 | ± | 2.34 | 20.83 | ± | 10.14*# | * |
| *Hspd1* | 1.00 | ± | 0.07 | 1.01 | ± | 0.18 | 1.03 | ± | 0.29 | 0.89 | ± | 0.19 | 1.30 | ± | 0.17& | 0.84 | ± | 0.16# | NS |
| *Lonp1* | 1.06 | ± | 0.39 | 1.25 | ± | 0.83 | 1.08 | ± | 0.44 | 0.89 | ± | 0.31 | 1.05 | ± | 0.38 | 0.61 | ± | 0.31 | NS |
| *mt-Atp6* | 1.15 | ± | 0.69 | 1.06 | ± | 0.40 | 1.02 | ± | 0.26 | 0.58 | ± | 0.20 | 1.62 | ± | 0.42& | 1.77 | ± | 0.73& | NS |
| *mt-Co2* | 1.15 | ± | 0.66 | 1.11 | ± | 0.53 | 1.03 | ± | 0.31 | 0.58 | ± | 0.19 | 1.51 | ± | 0.38& | 1.09 | ± | 0.22 | NS |
| *mt-Nd1* | 1.12 | ± | 0.61 | 1.08 | ± | 0.48 | 1.03 | ± | 0.29 | 0.43 | ± | 0.15 | 1.23 | ± | 0.43& | 0.98 | ± | 0.36 | NS |
| *Ndufs3* | 0.87 | ± | 0.23 | 1.01 | ± | 0.13 | 1.10 | ± | 0.41 | 0.75 | ± | 0.20 | 0.98 | ± | 0.23 | 0.78 | ± | 0.21 | NS |
| *Oma1* | 0.86 | ± | 0.25 | 1.08 | ± | 0.44 | 1.69 | ± | 1.34 | 0.80 | ± | 0.21 | 0.46 | ± | 0.22 | 0.26 | ± | 0.28* | * |
| *Ppargc1a* | 1.08 | ± | 0.47 | 1.17 | ± | 0.66 | 1.45 | ± | 0.83 | 0.65 | ± | 0.08 | 0.71 | ± | 0.46 | 0.15 | ± | 0.07* | * |
| *Rfesd* | 1.07 | ± | 0.42 | 1.03 | ± | 0.24 | 1.15 | ± | 0.49 | 0.75 | ± | 0.21 | 1.27 | ± | 0.44 | 1.31 | ± | 0.44 | NS |
| *Sdha* | 1.08 | ± | 0.44 | 1.02 | ± | 0.24 | 1.09 | ± | 0.41 | 0.71 | ± | 0.24 | 1.29 | ± | 0.42 | 0.98 | ± | 0.38 | NS |
| *Sdhb* | 1.12 | ± | 0.56 | 1.04 | ± | 0.34 | 1.14 | ± | 0.49 | 0.77 | ± | 0.17 | 1.23 | ± | 0.38 | 1.26 | ± | 0.40 | NS |
| *Sln* | 0.84 | ± | 0.29 | 1.11 | ± | 0.58 | 1.23 | ± | 0.86 | 141.07 | ± | 34.42 | 1009.51 | ± | 349.55*& | 1089.59 | ± | 786.50*& | * |
| *Spg7* | 1.06 | ± | 0.36 | 0.82 | ± | 0.19 | 1.50 | ± | 0.93 | 0.66 | ± | 0.13 | 0.69 | ± | 0.26 | 0.65 | ± | 0.30* | * |
| *Yme1l1* | 1.06 | ± | 0.36 | 1.09 | ± | 0.46 | 1.48 | ± | 1.06 | 0.83 | ± | 0.24 | 0.65 | ± | 0.33 | 0.53 | ± | 0.26* | * |
